# Habitat risk assessment for regional ocean planning in the U.S. Northeast and Mid-Atlantic

**DOI:** 10.1371/journal.pone.0188776

**Published:** 2017-12-20

**Authors:** Katherine H. Wyatt, Robert Griffin, Anne D. Guerry, Mary Ruckelshaus, Michael Fogarty, Katie K. Arkema

**Affiliations:** 1 Natural Capital Project, Stanford University c/o School of Environmental and Forest Sciences, University of Washington, Seattle, Washington, United States of America; 2 Natural Capital Project, Woods Institute for the Environment, Stanford University, Stanford, California, United States of America; 3 Northeast Fisheries Science Center, National Oceanic and Atmospheric Administration, Woods Hole, Massachusetts, United States of America; Hawaii Pacific University, UNITED STATES

## Abstract

Coastal habitats provide important benefits to people, including habitat for species targeted by fisheries and opportunities for tourism and recreation. Yet, such human activities also can imperil these habitats and undermine the ecosystem services they provide to people. Cumulative risk assessment provides an analytical framework for synthesizing the influence of multiple stressors across habitats and decision-support for balancing human uses and ecosystem health. To explore cumulative risk to habitats in the U.S. Northeast and Mid-Atlantic Ocean Planning regions, we apply the open-source InVEST Habitat Risk Assessment model to 13 habitats and 31 stressors in an exposure-consequence framework. In doing so, we advance the science priorities of EBM and both regional planning bodies by synthesizing the wealth of available data to improve our understanding of human uses and how they affect marine resources. We find that risk to ecosystems is greatest first, along the coast, where a large number of stressors occur in close proximity and secondly, along the continental shelf, where fewer, higher consequence activities occur. Habitats at greatest risk include soft and hard-bottom nearshore areas, tidal flats, soft-bottom shelf habitat, and rocky intertidal zones—with the degree of risk varying spatially. Across all habitats, our results indicate that rising sea surface temperatures, commercial fishing, and shipping consistently and disproportionally contribute to risk. Further, our findings suggest that management in the nearshore will require simultaneously addressing the temporal and spatial overlap as well as intensity of multiple human activities and that management in the offshore requires more targeted efforts to reduce exposure from specific threats. We offer a transparent, generalizable approach to evaluating cumulative risk to multiple habitats and illustrate the spatially heterogeneous nature of impacts along the eastern Atlantic coast and the importance of spatial scale in estimating such impacts. These results offer a valuable decision-support tool by helping to constrain the decision space, focus attention on habitats and locations at the greatest risk, and highlight effect management strategies.

## Introduction

Coastal and marine ecosystems sustain and enhance human well-being by providing important services such as protection from storms and erosion, habitat for economically important fish and shellfish species, high quality food resources, and opportunities for recreation, tourism, and inspiration [1–3]. For example, coastal habitats reduce storm exposure for over half the U.S. population [4]; globally, fisheries and aquaculture produce nearly US$150 billion in export value and provide over 20% of dietary protein for three billion people [5]; and travel and tourism—much of it dependent on ecosystem attributes—make up nearly 10% of global GDP [6]. Roughly a third of the human population lives within 100km of the coast today [7,8] and coastal and marine environments will become increasingly important as human population approaches 10 billion [9]. Yet, as the coastal population swells, these ecosystems face increasing anthropogenic threats related to energy and resource extraction, transportation, food security, recreation and tourism, coastal development, and climate change [10–12], imperiling their ability to provide services to current and future generations.

To address this challenge, managers, scientists, governments, and various stakeholder groups have called for ecosystem-based management (EBM) to understand multiple human uses, determine how these uses affect marine resources, and holistically manage marine and coastal species and habitats to secure the services they provide to people [10,13–17]. EBM builds on and synthesizes ecological, oceanographic, climate, and social science to develop and evaluate strategies that restore and sustain ecosystem function. For example, fisheries management is increasingly moving from a focus on single species towards ecosystem-based fisheries management that emphasizes linkages between and within ecosystems and places fisheries in the context of other uses of marine and coastal systems [16,18–20]. In parallel, ecosystem science has advanced to a point where it is possible to explicitly link changes in the state of ecosystems—resulting from management actions or climate change—to changes in services and the values that they provide across a wide array of ecosystems and service types [21–24]. Effective EBM serves to strengthen the delivery of ecosystem services by protecting ecosystems and understanding and manage multiple uses [25].

One important application of EBM is ocean planning, a process of assessing and allocating human activities to achieve multiple objectives in marine areas. Globally, more than three dozen countries are involved in, or have completed, some form of marine spatial planning [26,27]. For example, the European Union has been a leader in moving from single-sector management approaches to ecosystem-based, holistic environmental management [28,29]. Scientists and resource managers around the Great Barrier Reef Marine Park in Australia have collaborated to implement a comprehensive marine spatial plan with effective monitoring, evaluation, and adaptive management that emphasizes the entire seascape over any individual reef [30,31]. In the Caribbean, Belize recently adopted an innovative, integrated coastal zone management plan to balance development and conservation for a public-driven vision of the future [23,32]. And in the United States, National Ocean Policy has similarly embraced ocean planning to steward the oceans, coasts, and Great Lakes to support well-being and prosperity [33]. This Policy highlights science as the foundation of improved management and supports nine Regional Planning Bodies to coordinate information sharing and planning. To date, this has resulted in collaborative marine planning between Federal, State, and Tribal governments and the release of regional ocean plans and associated data portals for the Northeast and the Mid-Atlantic [34,35].

A key tension that EBM and ocean planning processes must address is that human activities in the ocean and coastal zone, while they enhance human well-being in the short term, may simultaneously compromise the functioning of ecosystems that underpin this well-being in the long-term. Risk assessment, which is based on components of exposure (or occasionally, ‘probability’) and consequence (or ‘severity’ or ‘sensitivity’) offers a comprehensive and quantitative methodology to assess the combined influence of multiple human activities and natural stressors on the ecosystem. Risk assessment provides managers with needed decision-support for ocean planning by helping to prioritize management areas and effective management strategies. It has become a fundamental component of EBM [36–43] and has been incorporated into the U.S. National Oceanic and Atmospheric Administration (NOAA)’s Integrated Ecosystem Assessments [36,37,44,45].

This analysis is the first to assess habitat risk from a diverse set of human activities and natural stressors across the entirety of the U.S. Northeast and Mid-Atlantic planning areas in the National Ocean Policy, incorporating the extensive human use and ecosystem mapping efforts conducted as part of the recent regional planning processes. We build on previous examples of risk assessment in this region that were focused on climate stressors [43], social vulnerability of fishing communities [46], and cumulative impacts to habitats in the state waters of Massachusetts [47], offering a transparent, flexible, and replicable approach and decision-support tool for iterations into the future. In this study, our objectives are to identify areas of high ecosystem risk across the regions, determine which habitats are at the greatest risk and where, and determine the primary causes of risk across habitats, locations, and spatial scale. To demonstrate the utility of these results, we show how this spatial risk assessment approach can be used in a management context to assess tradeoffs in siting new uses. In addition, we highlight management strategies that might best mitigate risk by identifying habitats where risk is caused by multiple stressors with relatively low consequence (thereby suggesting integrated management is needed) and those where risk is caused by a few, highly consequential stressors (thereby suggesting a targeted approach would be most effective).

## Materials and methods

### Study area

We explore the risk of habitat degradation from human activities along the Atlantic coast of the United States, focusing on two regions of ocean governance: The Northeast region that stretches from Maine to Connecticut, and the Mid-Atlantic region that continues from New York to Virginia. These regions are the first two areas along the coast of the United States to establish Regional Planning Bodies (RPBs) under the U.S. National Ocean Policy. These areas critically rely on adjacent marine habitats for livelihoods and well-being: in 2013, economic activity connected to the ocean in these states represented 935 thousand jobs and $63 billion in GDP [48]. Successful marine spatial planning efforts at the state level, such as those in Rhode Island [49] and Massachusetts [47] provided the preconditions for the U.S. Northeast to establish the first RPB, followed soon thereafter by the Mid-Atlantic RPB. The planning processes for these regions resulted in final ocean plans in December of 2016 that focus on ecosystem health, data collection (including extensive data portals), compatibility among uses, and effective decision making [34,35]. These plans are living documents, supported by ongoing engagement by the RPBs and supporting institutions, including the Northeast Regional Ocean Council (NROC) and the Mid-Atlantic Regional Council on the Ocean (MARCO), and are meant to provide actionable information to coastal decision-makers on an ongoing basis.

In this paper, we advance the science priorities of the Northeast and Mid-Atlantic regions by synthesizing the wealth of human use data available on regional portals and then assessing the impact these uses have on marine ecosystems. The Northeast Ocean Plan details an array of science priorities, including a priority to advance EBM and to improve our understanding of human uses and how these uses affect marine resources. The Mid-Atlantic plan echoes these priorities, and emphasizes cataloguing and synthesizing human uses through the Human Use Data Synthesis project [50]. Moreover, this work will provide spatial anthropogenic ecosystem risk data for NOAA’s Northeast U.S. Continental Shelf Integrated Ecosystem Assessment Program, in particular as part of its Ecosystem Status Report effort to characterize baseline ecosystem condition and ecosystem service provision across our study area.

### Estimating risk

To assess the influence of multiple human activities on coastal and marine ecosystems, we apply the InVEST Habitat Risk Assessment (HRA) model. The HRA model is a quantitative approach to evaluating the cumulative influence of stressors associated with human activities on habitats [23,51] and is available as part of the open-source InVEST ecosystem service modeling software [52]. HRA uses a well-established approach [51,53–55] from the risk literature that originates from fisheries vulnerability assessment [37,39,56] and was subsequently adopted for ecological risk assessment [23,51]. A central feature and strength of the HRA model is that it explicitly breaks down exposure and consequence in a risk framework and, in so doing, offers insight into effective management strategies. To assess the exposure of ecosystems to activities and the consequence of that exposure, synthesizes information about interactions between multiple human activities and multiple habitats using both spatial and non-spatial data from the peer-reviewed literature, grey literature, and expert opinion. Below, we explain the concept of exposure and consequence, the specific criteria we used in this analysis, where we derived scores for each criterion, and then walk through how these pieces fit together to estimate risk.

The HRA model assesses the risk of habitat degradation from each stressor based on several exposure and several consequence criteria [51]. *Exposure* is the degree to which a habitat encounters a stressor and *consequence* is the habitat-specific result of that exposure. Both exposure and consequence are made up of multiple criteria that can be defined by the user. In this analysis, exposure criteria include spatial data of the footprint (i.e. extent) and intensity of the stressor as well as non-spatial information about the frequency of occurrence and scale of effect of the impact (see Supporting Information and Table A in S1 File for additional details). Consequence criteria include change in biomass, trophic impact, and expected recovery time (see Supporting Information and Table A in S1 File for additional details). For each habitat-stressor combination, the model includes unique information (a.k.a., ‘scores’) for each exposure and consequence criteria. As an example of one score, we specify in the model that for seagrass beds, the recovery time is longer after dredging than recreational boating (see the data repository for the full compilation of scores).

We use the scores developed through the Massachusetts cumulative impact assessment (detailed in Kappel et al. [57]), which was based on expert elicitation, and modify them for use in the HRA exposure and consequence framework. This expert elicitation asked scientists to assess, for each cumulative impacts criterion (which match those exposure and consequence criteria explained above), the effect of each stressors on their habitat of expertise. Although inputs to the Habitat Risk Assessment model are traditionally informed by peer-reviewed literature, the breadth of this analysis in the Northeast and Mid-Atlantic (i.e., 13 habitats and 31 stressors) made this particularly challenging. Further, the use of scores from Kappel et al. [47] allows for the two approaches to be compared and used together. Past comparisons of scores between Massachusetts and California [57,58] demonstrate the transferability of scores beyond the area for which they were originally derived and provide us with the confidence to extrapolate cumulative impacts scores from Massachusetts to the broader Atlantic coast. We modify Kappel et al. [57] by weighting the components of exposure and consequence equally (as described in Eq 3) and by binning their continuous scores into three categories. This approach formats the scores for HRA and improves interpretability while preserving the relative rank of scores and the relative importance of high scores (see S1 File for additional details).

We use these scores—for each criterion of exposure and consequence for each habitat-stressor combination—in the model to estimate cumulative risk for each habitat and across ecosystems. Here we use a 1km cell size as the spatial processing unit and resolution of habitat risk outputs based on a conservative consideration of the resolution of the human activity inputs (Table C in S1 File). We first average (1) the set of exposure criteria and (2) the set of consequence criteria for each habitat-stressor combination (Eqs 1 & 2):

$$E_{jk}=\frac{\sum_{i=1}^{N}e_{ijk}}{N}\;C_{jk}=\frac{\sum_{i=1}^{N}c_{ijk}}{N}$$ (Eqs 1 & 2)

where E_jk_ is the exposure score specific to habitat *j*, from stressor *k;* C_jk_ is consequence score, *e*_*ijk*_ is the exposure rating criterion *i*, specific to habitat *j* and stressor *k; c*_*ijk*_ is the consequence rating. When the data were available (Table C in S1 File), we incorporated spatial variation in intensity into exposure. These equations result in a single exposure score and single consequence score for each habitat-stressor combination.

We then estimate risk associated with each habitat-stressor combination by calculating Euclidean distance to the origin, where the average exposure score is on one axis and the average consequence score is on the other (Eq 3):

$$R_{jkl}=\sqrt{{\left(C_{jkl}-1\right)}^{2}+{\left(E_{jkl}-1\right)}^{2}}$$ (Eq 3)

where risk, R_jkl_, to habitat *j* caused by stressor *k* in each location (cell) *l* is the Euclidean distance from the origin in an exposure-consequence plot. We map this habitat-stressor specific risk score where the habitat and stressor overlap in space (based on data presented in Tables B-C in S1 File and described in ‘habitats’ and ‘human activities and stressors’ sections below).

Cumulative risk to each habitat is the sum of the risk scores related the human activities co-occurring in each location containing that habitat:

$$R_{jl}=\sum_{k=1}^{K}R_{jkl}$$ (Eq 4)

Ecosystem risk for each grid cell is the sum of habitat risk scores in that cell:

$$R_{l}=\sum_{j=1}^{J}R_{jl}$$ (Eq 5)

Ecosystem risk (Eq 5) and cumulative risk to each habitat (a.k.a. ‘habitat risk’; Eq 4) provide the foundation of the results outlined in corresponding results sections, ‘Ecosystem Risk’ and ‘Habitat-Specific Risk’. We use a Tukey honest significant difference test with 95% family-wise confidence level to evaluate inter-regional differences in habitat risk (computed using HSD.test in the agricolae package for R). In addition, we use the breakdown of exposure and consequence within the risk equation (Eq 3) to inform the ‘Causes of Risk’ section of the results.

### Habitats

To map risk to habitats, we first classify the Atlantic coastal area—from Maine to Virginia—into 13 habitat types, ranging from nearshore habitats to deeper, offshore zones. To build on and to allow for comparisons with previous work in the region [47,57], we use the habitat classification scheme from the cumulative impact assessment of Massachusetts waters. We include eight nearshore habitats—beaches, tidal flats, salt marsh, rocky intertidal, seagrass, algal, nearshore soft-bottom, nearshore hard-bottom—and five offshore habitats—soft-bottom shelf, hard-bottom shelf, bathyal shelf (waters deeper than 200m), shallow pelagic, and deep pelagic. Spatial data for these habitats come from a combination of trusted regional data portals (i.e. Northeast Ocean Data and Mid-Atlantic Ocean Data portals) and federal data sources (i.e. NOAA’s Environmental Sensitivity Index, National Wetlands Inventory, NOAA’s U.S. Coastal Relief Model, U.S.G.S Continental Margin mapping of sediment). As necessary and in order to use the most recent data and ensure full spatial coverage, we merge data from multiple sources. In the case of habitats defined by sediment and depth (e.g. soft- and hard-bottom nearshore and shelf), we intersect sediment and depth data to define the spatial extent. The use of recent, reliable, and publically available data allow for replication in new locations and adaptive management as data improve (see S1 File, Table B in S1 File, and Fig A in S1 File for additional details on these data).

### Human activities and stressors

To assess the cumulative risk of human activities to coastal and marine habitats in the region, we incorporate the impact of 31 stressors. It is standard practice in the risk-assessment community to use the term “stressor,” which is a value-laden term. It is important to recognize that the human activities represented in this analysis as “stressors” also enhance people’s lives by providing food, recreational opportunities, livelihoods, and more. The approach we present here acknowledges that the activities used to derive benefits can also adversely affect the very habitats that are the source of these benefits to people.

Using Kappel et al.’s analysis [47] as a starting point, we include stressors available in regional and national data portals and those specified as important by regional planning bodies. We roughly group these stressors into six themes: (1) fishing and aquaculture, (2) human structures, (3) coastal and marine uses/impacts, (4) land-based impact, and (5) climate change (Table C in S1 File). Within the fishing and aquaculture theme, we coalesce fishing into five discrete categories of use: artisanal, non-destructive demersal fishing, destructive demersal fishing, pelagic, and recreational, based on similarity of impact. Aquaculture includes three categories consisting of finfish predators, shellfish, and marine plants (Fig B in S1 File). The second theme, human structures, includes benthic structures, coastal engineering, and three types of energy-related structures (wind, liquid-natural gas, and tidal; Fig C in S1 File). For the third theme, the most inclusive, we categorize additional marine uses and impacts into human trampling, dredging, military activity, shipwrecks, ocean dumping, ocean mining, oil spills, shipping, and five types of tourist activities (wildlife viewing, recreational boating, surfing, kayaking, and SCUBA; Figs D-F in S1 File). The fourth theme, land-based impacts, includes power plants, inorganic, sediment, nutrient, and light pollution (Fig G in S1 File). Finally, to explore climate change, the last theme, we capture the relative rate of increasing sea surface temperatures based on temperature anomalies relative to historical means (Fig H in S1 File). While other measures of climate change are important, we focus on sea-surface temperature because it affects all included habitat classifications, these effects are well documented and understood relative to other measures of climate change (e.g., increasing UV light), and the spatial data are readily available (see S1 File, Table C in S1 File, and Figs B-H in S1 File for additional details on these data).

We selected data for this analysis to maximize reliability and reproducibility (see Table B in S1 File). Most data come from regional portals or federal sources, are publically available (with the exception of commercial fishing), are fewer than five years old (with the exception of nutrient loading and coastal engineering for some states), and have full spatial coverage across our area of interest (with the exception of aquaculture). For many human activities we use the data as provided; in other instances, we merge multiple data sets to create complete spatial coverage, add spatial buffers following Kappel et al.’s [47] protocols, convert raster to vector for use in the model, and/or bin spatial variation to define categorize intensity (see S1 File and Table B in S1 File for additional details). In the absence of consistently reported observed data throughout the study region, we model inorganic pollutions (using impervious surfaces as a proxy for non-point source pollution) and nutrients loading (using the USGS SPARROW model [59]) from upland land-use to coastal pour-points and estuaries (see Supporting Information).

The data reveal spatial variation in the intensity of several stressors. As applicable and as data allow, we model spatial variation in intensity for each stressor. To do this we assign the lowest quartile of use or impact (e.g., fishing effort, nutrient loading) the lowest intensity and the upper quartile the highest intensity, with the middle quartiles composing moderate intensity. This spatially-explicit intensity score is included as an exposure criterion in the risk equation (Eq 1). See the S1 File for additional details on this approach and how it was applied to various stressors.

## Results

### Ecosystem risk

**Fig 1:**
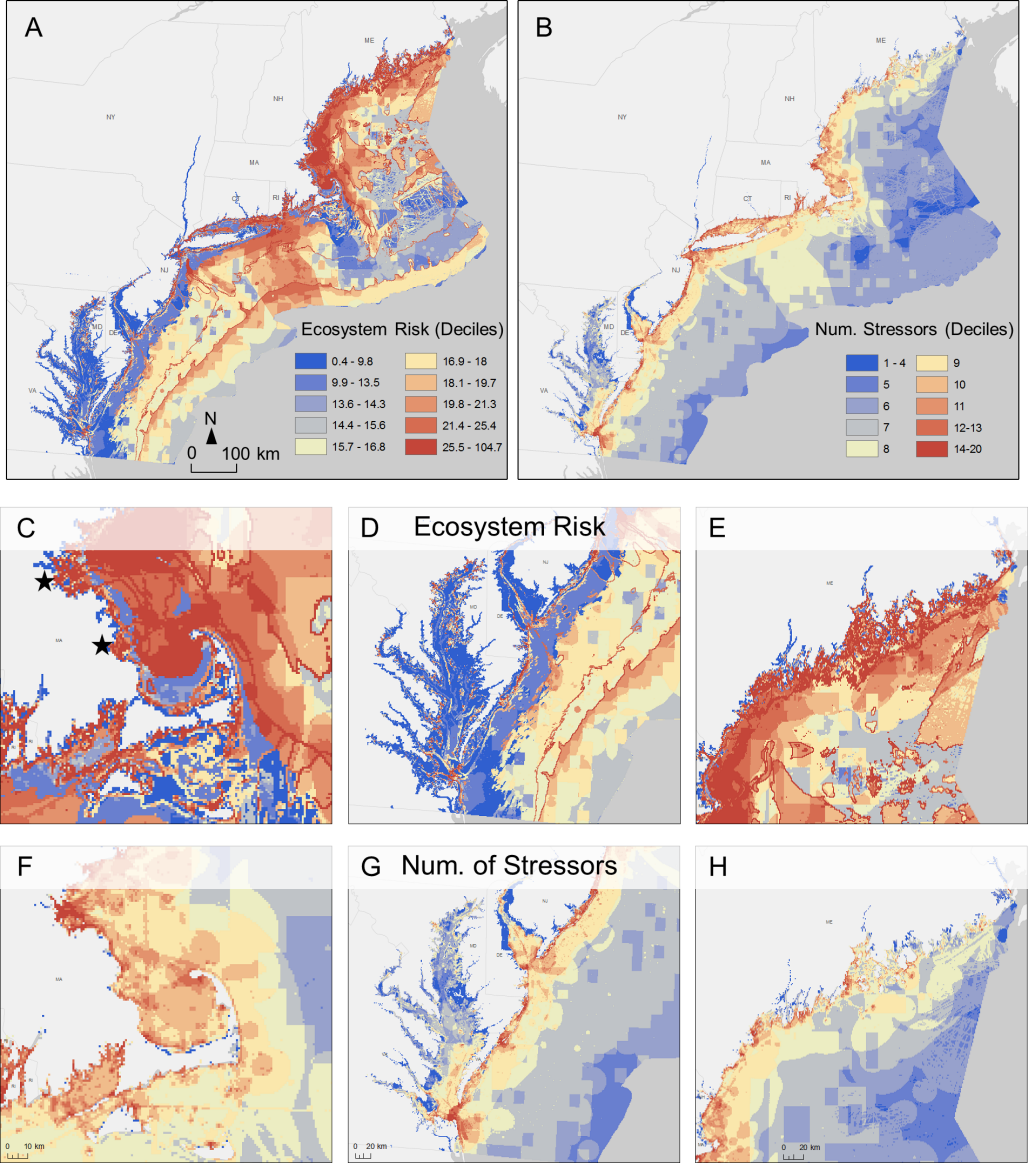
Ecosystem risk and overlapping stressors for the Northeast and Mid-Atlantic at different scales. In all maps, warmer colors represent greater risk or a greater number of overlapping human activities. (A) Ecosystem risk for the entire study area. Median risk is 15.8. (B) Number of overlapping stressors for the entire study area. The number of stressors is broken down by decile to allow for comparison with risk deciles. (C) Ecosystem risk map for Cape Cod, Massachusetts. The northern star is Boston Bay and the southern star is Plymouth Bay. (D) Ecosystem risk map for Delaware and Chesapeake Bay. (E) Ecosystem risk map for the Maine coastline. (F) Number of overlapping stressors for Cape Cod, Massachusetts. (G) Number of overlapping stressors for Delaware and Chesapeake Bay. (H) Number of overlapping stressors for the Maine coastline.

The highest levels of risk are concentrated along the coastline and along the continental shelf, though all areas throughout the Northeast and Mid-Atlantic region experience some degree of risk (Fig 1A). Indicating a skewed distribution of risk in which fewer areas are at particularly high risk, ecosystem risk ranges from 0 to 106 across both regions, with a median risk score of 15.8 and the upper quartile of risk at 18.7. Areas of particularly high risk occur along the entirety of the coastline. However, this zone of high coastal risk visible across both regions varies at finer spatial scales. For example, high risk areas occur in wider swaths along the coastlines of Delaware, Massachusetts, New Hampshire, and Maine and occur in a narrower, more variable stretch along the coastlines of Virginia and Maryland (Fig 1)[60]. A second area of higher risk is a 40 to 100-km wide zone at the edge of the continental shelf, stretching from a distance of 125km offshore (at the Virginia-North Carolina border in the south) to 425km (off the coast of Maine in the north; Fig 1A). Areas of lower risk (less than the median of 15.8) occur primarily in the Mid-Atlantic from distances just beyond the high-risk coastline (from 5 to 20km off shore) (Fig 1A).

The risk assessment approach provides spatially explicit information that highlights variation in in risk at multiple scales. While greater risk in the nearshore may be an intuitive result, our results show that nearshore risk is generally lower and less concentrated in the Mid-Atlantic than in the Northeast. Within the Mid-Atlantic, high risk areas are concentrated in northern New Jersey and at the mouths of the Delaware and Chesapeake Bays, while the rest of the Mid-Atlantic coastline is at lower risk. The Northeast coastline contains greater area at the highest risk levels, with risk concentrated along the coastlines of Massachusetts, New Hampshire, and Maine (Fig 1). The high risk area extends further offshore throughout the Gulf of Maine and Long Island Sound than it does along other sections of the coast. Illustrative of variation at the state scale, ecosystem risk is highest in Boston and Plymouth Bays (Fig 1C). And within a single bay, for example Cape Cod, risk is greater off the tip of the Cape Cod and along the coast than in the interior bay (Fig 1D). Beyond the nearshore area, areas of risk are even more spatially variable, with higher risk patches east of Long Island, along the northern extent of the U.S. continental shelf, and sporadically through the Gulf of Maine. Each of these spatial scales—comparable to regional, state, and bay-wide planning processes—reveals areas of particular concern and adds detail to our general understanding of coastlines at risk.

Ecosystem risk maps, which incorporate habitat locations as well as the exposure and consequence of each stressor on each habitat, go beyond simple un-weighted overlays of human activities and identify different areas (Fig 1). While overlay analysis illuminates the nearshore area as a place of potential conflict, it misses the especially high-risk waters (i.e. top decile) of Massachusetts and Maine, where the multitude of coastal habitats in close proximity suggest additional attention by managers may be needed. Risk analysis also highlights the high-risk offshore areas (Fig 1A) where there are few overlapping stressors (Fig 1B), demonstrating the relative importance of fewer, higher consequence stressors in the offshore area. Conversely, for areas 5 to 45km offshore (just beyond the immediate high risk coastline), and especially in the southern Mid-Atlantic, risk is lower (Fig 1A and 1D) than the number of activities would suggest (Fig 1B and 1G) because local activities are less impactful. Differences also emerge at finer spatial scales, for example in Delaware and Chesapeake Bays. While a simple overlay of stressors highlights much of Delaware Bay as a zone of many activities (Fig 1G), our cumulative risk analysis suggests the center of the bay faces the greatest threat (Fig 1D) resulting from high consequence activities like shipping and dredging. In the Chesapeake Bay, risk analysis highlights the importance of small bays and tributaries more than the simple overlay of stressors does (Fig 1D and 1G), reflecting the importance of land-based stressors in this bay. Patterns of population density along the coast [18] are evident in both sets of maps, but the overlay of human activities and risk analysis tell an increasingly rich story. Risk analysis provides more information than population density or overlap analysis because it includes the distribution of habitats as well as interactions between habitats and stressors. The following sections further explore the added value of these components.

### Habitat-specific risk

Patterns of risk further vary between habitats and across regions. The RPBs did not formally delineate boundaries between the Northeast and Mid-Atlantic planning areas, choosing to have some overlap in the waters off of New York. For the purposes of quantitative comparison between regions, we bisect the two planning areas with a line running from New York Harbor to the continental shelf near Hudson Canyon. This delineation is consistent with other research efforts supported by the RPBs under the National Ocean Policy [61].

**Fig 2:**
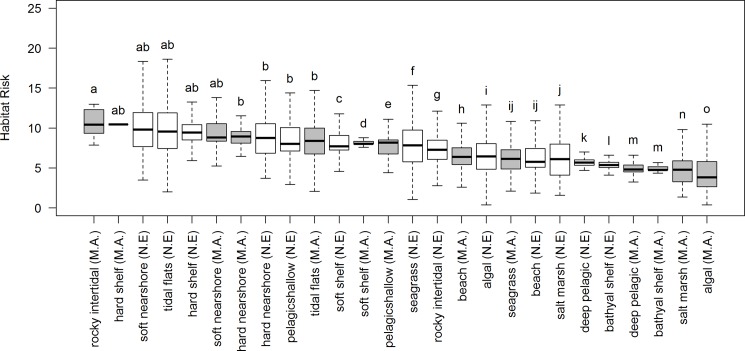
Distribution of cumulative risk for each habitat in each region. Boxes represent 25^th^ to 75^th^ percentiles, the bar represents the median, and dashed lines represent minimum and maximum values, having excluded outliers. Gray boxes represent habitats in the Mid-Atlantic and white boxes represent habitats in the Northeast. Letters represent groups of habitats that have statistically similar means from a Tukey honest significant difference test with a 95% family-wise confidence level.

Across regions, risk is highest for rocky intertidal, hard-bottom shelf, tidal flats, and soft and hard-bottom nearshore habitat. Higher median scores for these habitats indicate a greater area at high risk. Between regions, habitats are generally at greater risk in the Northeast than in the Mid-Atlantic. Risk is higher in the Northeast for tidal flat, seagrass, marsh, algal, soft-bottom nearshore habitat, soft-bottom shelf habitat, shallow and deep pelagic, and bathyal shelf habitats. Habitat risk is higher in the Mid-Atlantic only for rocky intertidal, hard-bottom shelf, hard-bottom nearshore, and beach habitat (Fig 2). These inter-regional differences in habitat risk are significant for all habitats except soft- and hard-bottom nearshore, hard-bottom shelf areas, and tidal flats (based on a Tukey honest significant difference test with 95% family-wise confidence level). The variation in risk scores indicates that risk to particular habitats is not homogenous across the area of analysis. For example, the wide range of risk to tidal flats in both regions indicates spatial variation in risk, whereas the narrow range in risk to soft-bottom shelf area indicate relatively homogenous risk across the area of analysis (Fig 2).

**Fig 3:**
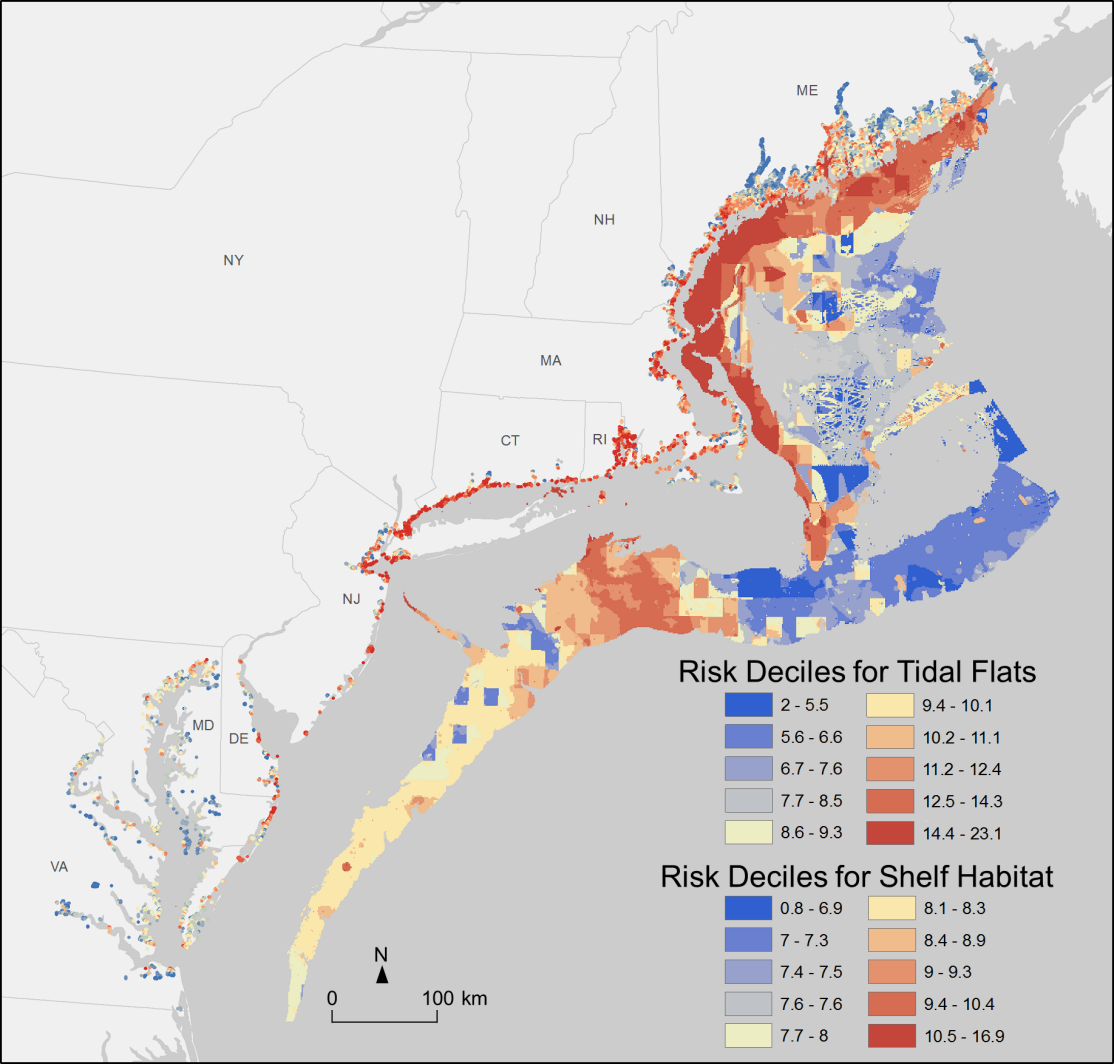
Habitat risk map for tidal flats and shelf habitats. Tidal flats occur along the coastline and shelf habitats are distant from the coastline. Note that risk deciles are scaled for each habitat independently. Shelf habitats include both soft and hard-bottom shelf habitat categories.

Tidal flats are one of the most at-risk habitats and this risk varies spatially. There are over 550sq. km of tidal flats in the Northeast and an additional 190sq. km in the Mid-Atlantic. Variation in risk is evident in the Northeast along the coastline of Connecticut, Rhode Island, and New Hampshire, where risk to tidal flats is predominantly in the upper quartile (quartile defined by both regions; Fig 3). In Maine, tidal flats south of Portland are at greater risk than those further to the north. Along the Massachusetts coast, tidal flats around Massachusetts Bay are at the greatest risk, while those along Cape Cod Bay and Nantucket Sound face lower risk. In the Mid-Atlantic, tidal flats at the greatest risk in Maryland along the northern section of Assateague State Park and in Virginia at the mouth of the Chesapeake Bay near Virginia Beach and Newport News (Fig 3). While the multitude of nearshore human activities affects risk to tidal flats everywhere, risk to these high risk areas is driven in particular by additional tourism (recreational boating, wildlife viewing, and SCUBA diving) as well as land-based stressors (e.g. power plants, nutrient loading, and light pollution), which do not occur in combination in lower risk areas. Given the importance of tidal flats for flood mitigation and fisheries, among other benefits [62], habitat risk maps offer an opportunity for regional, state, and bay-wide efforts to focus management towards specific locations.

Soft- and hard-bottom shelf habitat, often some of the most productive habitats in the ocean, are the offshore habitats most at risk. Hard-bottom shelf is the habitat most at risk after tidal flats and covers over 4,500sq. km in the Northeast and just 150sq. km in the Mid-Atlantic. Soft-bottom shelf habitat covers nearly 72,500sq. km in the Northeast and 22,230sq. km in the Mid-Atlantic. Mean risk values are similar between the two regions for both soft- and hard-bottom shelf (Fig 2). In the Northeast, these habitats are at the greatest risk in the interior Gulf of Maine; risk decreases with increasing distance from the coast. A second area of relatively high risk exists offshore off Long Island and Connecticut, with lower, more variable risk areas spanning to the north and to the south into the Mid-Atlantic (Fig 3). Risk in these locations is driven, in part, by the higher intensities of non-destructive demersal fishing in combination with recreational fishing, wildlife viewing, shipping, and rising sea surface temperatures.

### Causes of risk

**Fig 4:**
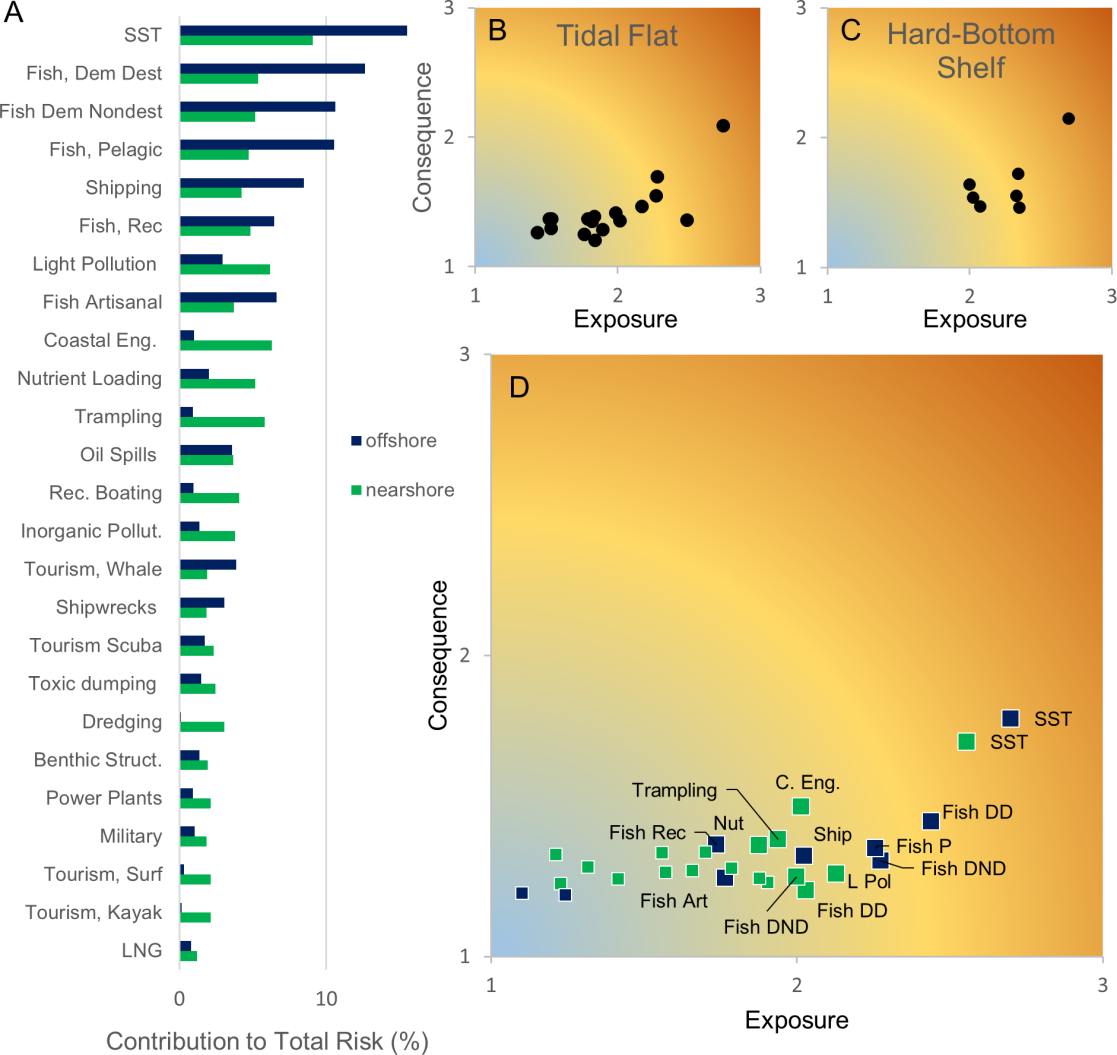
Causes of risk for nearshore and offshore habitats. (A) Relative contribution of different human activities and stressors to total risk for nearshore and offshore habitats types. The relative contribution is averaged across all nearshore or offshore habitats. Nearshore habitats include beach, salt marsh, tidal flats, rocky intertidal, algal, seagrass, soft-bottom nearshore, and hard-bottom nearshore habitats. Offshore habitats include soft-bottom shelf, hard-bottom shelf, bathyal shelf, shallow pelagic, and deep pelagic zones. (B) Risk plot for tidal flats and (C) hard shelf habitat (combined across Northeast and Mid-Atlantic); each point represents a unique human activity or stressor. For each habitat, included human activities (points) are cumulatively responsible for 80% of risk. (D) Risk plot for nearshore (green) and offshore (blue) habitats. Points represent the unique exposure and consequence from a particular stressor. Points shown for each habitat category cumulatively contribute 80% of risk. Individual human activities or stressors contributing more than 5% to risk are labeled (SST = increasing sea surface temperatures, Fish DD = demersal destructive fishing, Fish NDD = non-destructive demersal fishing, Fish A = Artisanal fishing, Tramp = human trampling, C Eng = coastal engineering).

Across habitats and locations, rising sea-surface temperatures and commercial fishing are the individual stressors responsible for the greatest amount of risk, yet the unique combination of stressors that affect categories of offshore and nearshore habitats as well as each individual habitat offer a more nuanced and informative explanation. For offshore habitats (depths > 30m; bathyal shelf, soft-bottom shelf, hard-bottom shelf, shallow pelagic, and deep pelagic), 75% of risk is explained by eight human activities: warming sea surface temperatures, four kinds of commercial fishing, shipping, recreational fishing, and wildlife viewing (Fig 4A). Specifically, for soft and hard-bottom shelf habitats, rising sea surface temperatures, four kinds of commercial fishing, and shipping collectively account for half to three-quarters of total risk across the area of analysis. In contrast to offshore habitats, for nearshore habitats (i.e., beaches, tidal flats, salt marsh, rocky intertidal, seagrass, algal, nearshore soft-bottom, nearshore hard-bottom), a larger suite of stressors contributes to risk; 15 stressors, each contributing a smaller percentage to overall risk, are collectively responsible for 75% of risk. Human trampling, light pollution, coastal engineering, and nutrient loading—in addition to the key stressors identified for offshore regions—each contribute more than 5% to risk, and many more contribute smaller percentages (Fig 4A). The greatest contributors to risk to tidal flats across both regions are warming sea surface temperatures, adjacent human populations (e.g. through trampling), coastal engineering, and land-based nutrient loading, sediment, and inorganic pollution; these seven stressors are collectively responsible for almost half the total risk to tidal flats. The effect of these stressors is evident and important in specific locations: the area of high risk off the Massachusetts, New Hampshire, and Maine coasts corresponds with the highest intensities of non-destructive demersal fishing; the area of high risk off of Long Island and Connecticut is associated with non-destructive demersal, artisanal, and pelagic fishing; areas of fine-scale low risk to the east of Cape Cod are associated with lower shipping density; and areas of low risk to the north, southeast, and south of Cape Cod are areas where fishing is restricted (Fig 3 and Supporting Information).

Risk can be further deconstructed to determine how habitat types are variably affected by the exposure and consequence of stressors (Fig 4B–4D). Of the 15 stressors (ranging from rising sea-surface temperatures to coastal engineering to trampling) that cumulatively account for 75% of risk to tidal flats, average exposure (i.e., from spatial extent, intensity, frequency of occurrence, and scale of effect) varies between these stressors, ranging from 1.52 to 2.74 (where 3 is the maximum exposure score). In contrast, the consequence (e.g., change in biomass, trophic impact, and recovery time) of these multiple stressors to tidal flats is consistently lower (ranging from 1.21 to 1.7 with one outlier at 2.1) (Fig 4B). Because each individual stressor results in a relatively minor change to habitat quality (i.e., has a low consequence score), reducing risk to tidal flats will require reducing exposure to a number of individual stressors (Fig 4B). In contrast, the eight stressors—from warming sea surface temperatures to fishing and shipping—that account for 75% of the risk to hard-bottom shelf habitat have greater exposure (e.g., from greater temporal and spatial overlap or higher intensity) and consequence (i.e., greater impact on habitat quality) (Fig 4C). Reducing exposure from a single stressor, either completely or by limiting overlap, intensity, or frequency of occurrence, will reduce risk by a relatively large amount for these habitats. Nearshore and offshore habitats follow a similar pattern to tidal flats and hard-bottom shelf, respectively, where risk to nearshore habitats is caused by a multitude of stressors with relatively low consequence, and risk to offshore habitats is caused by fewer stressors with greater exposure (Fig 4D). Exposure and consequence information can be used to improve habitat quality; reducing risk to nearshore habitats will require simultaneously addressing the frequency of occurrence and intensity of multiple human activities and reducing risk to offshore habitats requires more targeted efforts to reduce exposure from specific threats.

## Discussion

This analysis is, to our knowledge, the first to assess the cumulative risk to habitats across the entirety of the Northeast and Mid-Atlantic Ocean Planning regions. In doing so, we advance the science priorities of EBM and both regional planning bodies by synthesizing the wealth of available data to improve our understanding of human uses and how they affect marine resources. In this analysis, we identify areas of high ecosystem risk across the regions, investigate which habitats are at the greatest risk and where, and determine the primary causes of risk across habitats and locations, illustrating the effect of spatial scale on risk evaluations. We find that risk to ecosystems is greatest 1) along the coast, where a large number of stressors occur in close proximity and 2) in a relatively narrow outer offshore region, where fewer, higher consequence activities occur. Habitats at greatest risk include soft and hard-bottom nearshore areas, tidal flats, soft-bottom shelf habitat, and rocky intertidal zones—with the degree of risk varying spatially. Across all habitats, our results indicate that rising sea surface temperatures, commercial fishing, and shipping consistently and disproportionally contribute to risk. These results offer a valuable decision-support tool by helping to constrain the decision space, focus attention on habitats and locations at the greatest risk, and highlight effect management strategies.

**Fig 5:**
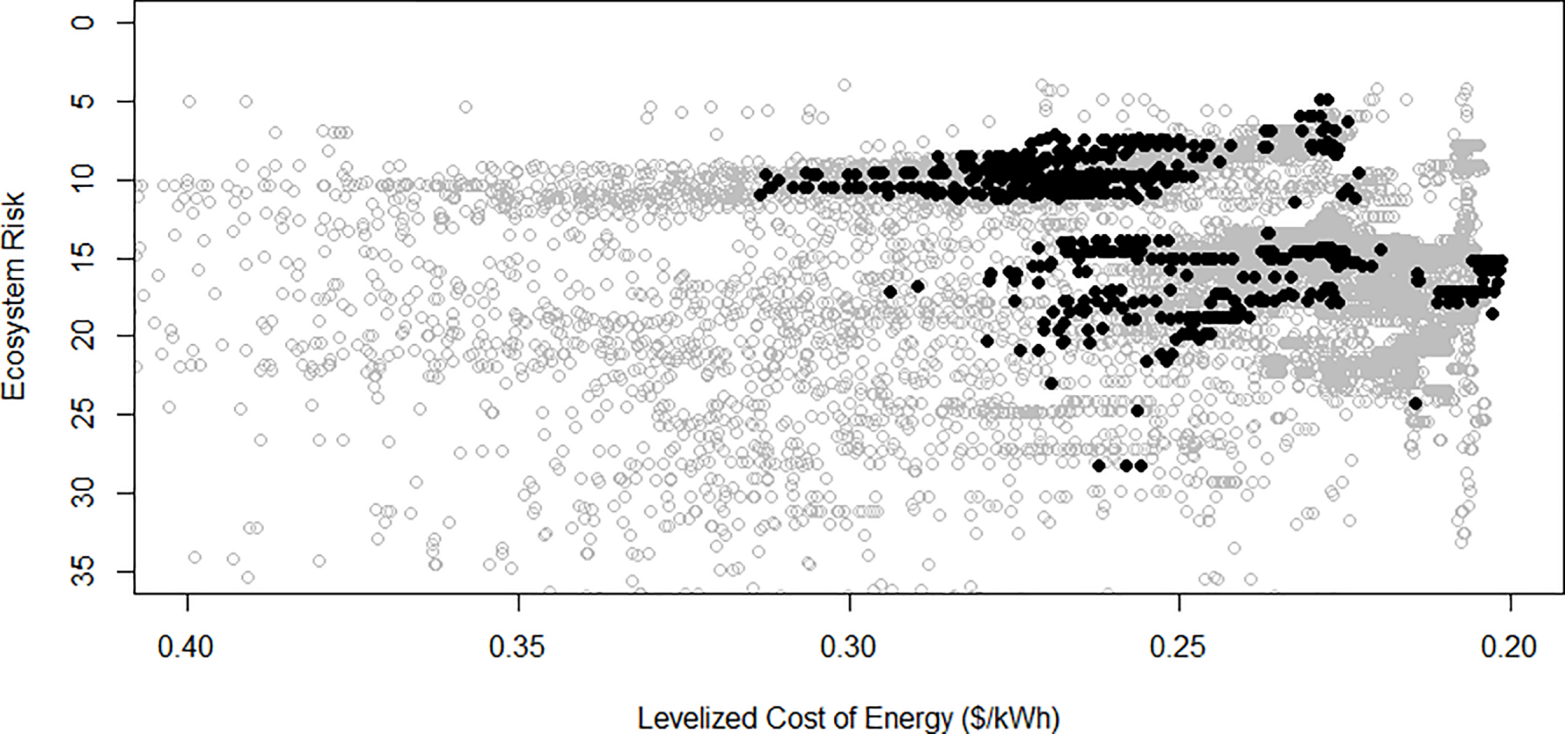
Inverse scatterplot of ecosystem risk and the levelized cost of energy for leased (black) and unleased (gray) locations throughout our study areas. The outer envelope of these points represents the *production possibility frontier* for siting a windfarm of 80 5-megawatt turbines in fewer than 60m water depth. Currently leased areas cluster near the minimum risk and minimum cost areas, but not around the joint minimum of both (upper right hand corner).

As the Northeast and Mid-Atlantic regional planning bodies begin implementing their ocean management plans, ecosystem risk maps can help constrain the decision space. These planning regions cover nearly a quarter of a million square kilometers and decision-makers within them are tasked with managing a multitude of activities, habitats, and interests. Specific agencies like the Bureau of Ocean Energy Management (BOEM), for example, could use risk maps in conjunction with spatially-explicit wind energy values to explore trade-offs between the two as they site new lease areas. As an example, we plot the levelized cost per unit of energy (‘levelized’ accounts for startup costs, discount rate, and continuous maintenance; see Supporting Information) for all potential wind energy sites in our study area against the current ecosystem risk in those locations (Fig 5). We overlay on this currently leased wind farm areas (black dots). In a retrospective sense, this analysis shows that existing lease areas are sited in locations that have low risk or low production costs, but not both; looking forward, it identifies new potential lease areas that can improve in both dimensions (upper right quadrant) located near the existing Rhode Island/Massachusetts lease area and off the coast of Virginia. This type of tradeoff analysis is flexible, in the sense that it can accommodate different siting preferences, such as a desire to site wind energy in high risk areas to concentrate risk into fewer areas, or different uses, such as advance screening for appropriate locations for the emerging offshore aquaculture industry.

Similarly, identifying which habitats are at the greatest risk and where provides an opportunity to focus management and restoration priorities. Our results show that tidal flats across the Northeast and Mid-Atlantic are at especially high risk and that the benefits this habitat provides—e.g. flood protection and fish nursery habitat important for commercial and recreational fishing—may also be threatened. Tidal flats along the coasts of Connecticut, Rhode Island, and New Hampshire are at the greatest risk and are at variable risk across Maine, Massachusetts, and the Mid-Atlantic (Fig 3); planners, for example NOAA’s Office of Coastal Management (OCM) or the Northeast Regional Planning Body Restoration Subcommittee, could use this spatial information to allocate resources or identify for further study. For example, OCM, along with [63] New Hampshire’s Department of Environmental Services Coastal Program and Eastern Research Group, are already using HRA to prioritize areas for restoration in New Hampshire’s Great Bay [63]. Habitat risk maps could also be overlaid with Essential Fish Habitat (e.g., as compiled on the Northeast Ocean Data portal) to identify where important fish habitat might be at risk and prioritize management of those areas. Identifying which habitats and locations are most at risk offers powerful data for managers facing a range of decisions.

In addition to identifying those areas and habitats most at risk, this risk assessment approach identifies those human activities and stressors causing the greatest amount of risk and could be used to direct management towards these human activities. Fishing and shipping were found to be primary causes of risk for all habitats—along with land-based nutrient increases, coastal engineering, and human trampling as causes of risk for nearshore habitats—and indicate the need for additional investigation and community outreach into the management of these activities. Making the case for moving beyond traditional single-sector management, the high consequence of rising sea surface temperatures on all habitats suggests that climate adaptation will need to be simultaneously addressed with management of any single human activity. Through the Ocean Planning Process, the Northeast and Mid-Atlantic Planning Bodies, armed with data about the most impactful stressors, have an opportunity to increase community outreach and coordination between key agencies in order to move towards integrated EBM.

Finally, the breakdown of risk by exposure and consequence highlights management strategies that best mitigate that risk. Simultaneously reducing multiple stressors with low consequence, as is required in the nearshore, might necessitate a more integrated, collaborative management approach. Alternatively, reducing a smaller subset of more consequential stressors, as is needed in the offshore area, suggests that a more targeted approach would be effective. For tidal flats specifically, our results show that risk from human trampling and coastal engineering (the most impactful stressors) are primarily the result of exposure rather than consequence; management that focuses on addressing total spatial extent, frequency, or intensity of these activities will better alleviate the effects of these activities than efforts to reduce the consequence of these activities. Identifying the root causes of risk is essential for helping agencies, from BOEM to NOAA to the RPBs, select effective management strategies.

In addition to providing management tools and insight, HRA is an integral step in connecting the multitude of stressors to changes in ecosystem services. In New Hampshire’s Great Bay, NOAA’s Office for Coastal Management and others [63], related current and estimated future risk to eelgrass, saltmarsh, and oyster beds to losses in recreational fishing, recreational oyster harvesting, and commercial aquaculture using a benefits-transfer approach with implications for restoration planning and aquaculture siting. In Belize, Arkema et al. [23], linked risk to mangrove, coral, and seagrass habitats to InVEST ecosystem service models to quantify how alternative scenarios proposed under the coastal planning process would affect coastal vulnerability from storms, lobster catch and revenue, and tourist visitation and expenditure. Stakeholders and the decision-makers then used habitat risk and ecosystem service outputs as the backbone of the new the Belize Integrated Coastal Zone Management Plan [32], ratified by the legislature in 2016. In the Northeast and Mid-Atlantic, the habitats we assess support the regional fishing economy (e.g., by providing nursery and adult habitat to lobster and commercial fish species), the cultural identity of coastal communities, the coastline’s resilience to storms and erosion (e.g., by attenuating waves and storing flood water), and the nature-based tourism industry (e.g., by providing opportunities for boating, wildlife viewing, and more). Future work could explicitly quantify impacts on ecosystem services, since those habitats most at risk—i.e., soft- and hard-bottom nearshore habitat, tidal flats, soft and hard-bottom shelf, and rocky intertidal zones (Fig 2)—may have reduced capacity to provide important services. This could be especially important where populated coastlines with degraded habitats are vulnerable to storms or where fishing grounds might shift with habitat quality [64].

This approach is, of course, imperfect. Four issues merit further discussion. First, HRA’s additive approach does not capture positive or negative dynamics between stressors or across time. In some cases, our additive approach to assessing risk will underrepresent risk by missing interactions between stressors that might be synergistic or over-represent those that might cancel one another out [65,66]. Further, HRA offers a snapshot in time and does not explicitly account for the historical impacts or ecological legacies (e.g., past fishing practices) of human activities. Second, the nature of cumulative risk means that results are best interpreted relative to one another [67]; uncertainty analysis has shown broad qualitative trends in this type of impact mapping to be robust [68]. Empirical testing of HRA elsewhere has shown strong relationships between modeled risk and habitat fragmentation and health [51]. As empirical data become available locally, a great avenue of future work would be to validate and relate regional risk scores to conditions of habitat quality (e.g., density, fragmentation, etc.). Third, results are conditioned on our choice of habitats, stressors, and area of interest; for example, we do not include oyster reefs or disease outbreaks and these components may be important in particular areas like the Chesapeake Bay. Finally, our application of the HRA approach in this instance is built upon subjective scores (i.e., derived from expert elicitation) for exposure of habitats to stressors and the consequences of those exposures. Fortunately, we could rely on earlier published work for the large number of habitat stressor combinations explored here, but where possible, we recommend using peer-reviewed literature to inform the inputs to HRA.

Despite these limitations, we believe our habitat risk assessment approach (and more specifically, the InVEST HRA model [51] as a means to operationalize it) supports the needs of regional ocean planning and EBM by providing a framework (and tool) to explore the cumulative effects of multiple human activities and stressors on marine and coastal habitats. The flexible and repeatable methodology we applied helps to synthesize and employ the volumes of data created and collected by scientists, organizations, and agencies in the U.S. Northeast and Mid-Atlantic in order to meet planning objectives. Further, the use of a spatially explicit cumulative risk assessment approach can help illuminate a sustainable balance between the beneficial use of coastal areas and the adverse impacts of that use. Specifically, our results help to (1) constrain the decision space for management by identifying areas at the greatest risk, (2) focus attention on habitats and locations at the greatest risk, and (3) highlight effective management strategies by identifying especially impactful stressors and isolating where integrated approaches for many, low-consequence stressors or targeted efforts towards a few consequential stressors would be most effective. Results can be iterated over time to respond to changes in climate and other stressors, as well as management’s response to them.

## Supporting information

S1 File Supporting information for methodology. Additional explanation and details for the methods and data used in this study including estimating risk, habitat data, human activities and stressor data, and the wind energy analysis. Table A, Exposure and consequence criteria used in the Habitat Risk Assessment model; Fig A, Habitat types; Table B, Habitat data and sources; Table C, Human activity and stressor data; Fig B, Spatial distribution of aquaculture of five kinds of fishing; Fig C, Human structures; Fig D, Human trampling, shipping, and shipwrecks; Fig E, Military danger zones, ocean dumping, sand and gravel mining, and oil spills; Fig F, Tourist activities; Fig G, Land-based stressors; Fig H, Increasing sea surface temperature.
(DOCX)

## References

[1] Peterson CH, Lubchenco J. Marine ecosystem services In: Daily G, editor. Nature’s Services: Societal Dependence on Natural Ecosystems. 4th ed. Washington, D.C.: Island Press; 1997 pp. 177–194.

[2] Barbier EB, Hacker SD, Kennedy C, Koch EW, Stier AC, Silliman BR. The value of estuarine and coastal ecosystem services. Ecol Monogr. 2011;81: 169–193. doi: 10.1890/10-1510.1

[3] Guerry AD, Ruckelshaus MH, Arkema KK, Bernhardt JR, Guannel G, Kim C-K, et al Modeling benefits from nature: using ecosystem services to inform coastal and marine spatial planning. Int J Biodivers Sci Ecosyst Serv Manag. 2012;8: 107–121. doi: 10.1080/21513732.2011.647835

[4] Arkema KK, Guannel G, Verutes G, Wood SA, Guerry A, Ruckelshaus M, et al Coastal habitats shield people and property from sea-level rise and storms. Nat Clim Change. 2013;3: 913–918. doi: 10.1038/nclimate1944

[5] The State of the world fisheries and aquaculture 2016: Contributing to food security and nutrition for all. Food and Agriculture Organization of the United Nations; 2016.

[6] Travel and Tourism Economic Impact 2016 World [Internet]. World Travel & Tourism Council; 2016. Available: https://www.wttc.org/research/economic-research/economic-impact-analysis/

[7] Small C, Nicholls RJ. A Global Analysis of Human Settlement in Coastal Zones. J Coast Res. 2003;19: 584–599.

[8] Intergovernmental Panel on Climate Change (IPCC). Climate Change 2014: Impacts, Adaptation, and Vulnerability. Working Group II Contribution to the IPCC 5th Assessment Report. IPCC; 2014.

[9] World Population Prospects: The 2015 Revision, Key Findings and Advance Paper [Internet]. New York: United Nations Department of Economic and Social Affairs, Population Division; 2015. Report No.: ESA/P/WP.241. Available: https://esa.un.org/unpd/wpp/

[10] Lubchenco J, Sutley N. Proposed U.S. Policy for Ocean, Coast, and Great Lakes Stewardship. Science. 2010;328: 1485–1486. doi: 10.1126/science.1190041 20558691 10.1126/science.1190041

[11] Halpern BS, McLeod KL, Rosenberg AA, Crowder LB. Managing for cumulative impacts in ecosystem-based management through ocean zoning. Ocean Coast Manag. 2008;51: 203–211. doi: 10.1016/j.ocecoaman.2007.08.002

[12] Millennium Ecosystem Assessment. Ecosystems and Human Well-Being: Synthesis. Island Press; 2005.

[13] Leslie HM, McLeod KL. Confronting the challenges of implementing marine ecosystem-based management. Front Ecol Environ. 2007;5: 540–548. doi: 10.1890/060093

[14] Paul LMB. The 2003 Pew Oceans Commission Report: Law, Policy, and Governance. Nat Resour Environ. 2004;19: 10–16.

[15] An Ocean Blueprint for the 21st Century. Washington, D.C: U.S. Commission on Ocean Policy; 2004. Report No.: 0–9759462–0–X.

[16] Fogarty MJ. The art of ecosystem-based fishery management. Can J Fish Aquat Sci. 2013;71: 479–490. doi: 10.1139/cjfas-2013-0203

[17] Santos CF, Agardy T, Andrade F, Barange M, Crowder LB, Ehler CN, et al Ocean planning in a changing climate. Nat Geosci. 2016;9: 730–730. doi: 10.1038/ngeo2821

[18] Samhouri JF, Haupt AJ, Levin PS, Link JS, Shuford R. Lessons learned from developing integrated ecosystem assessments to inform marine ecosystem-based management in the USA. ICES J Mar Sci J Cons. 2014;71: 1205–1215. doi: 10.1093/icesjms/fst141

[19] Fulton EA, Link JS, Kaplan IC, Savina-Rolland M, Johnson P, Ainsworth C, et al Lessons in modelling and management of marine ecosystems: the Atlantis experience. Fish Fish. 2011;12: 171–188. doi: 10.1111/j.1467-2979.2011.00412.x

[20] Salomon AK, Gaichas SK, Jensen OP, Agostini VN, Sloan N, Rice J, et al Bridging the Divide Between Fisheries and Marine Conservation Science. Bull Mar Sci. 2011;87: 251–274. doi: 10.5343/bms.2010.1089

[21] Harrison PA, Berry PM, Simpson G, Haslett JR, Blicharska M, Bucur M, et al Linkages between biodiversity attributes and ecosystem services: A systematic review. Ecosyst Serv. 2014;9: 191–203. doi: 10.1016/j.ecoser.2014.05.006

[22] Sandifer PA, Sutton-Grier AE, Ward BP. Exploring connections among nature, biodiversity, ecosystem services, and human health and well-being: Opportunities to enhance health and biodiversity conservation. Ecosyst Serv. 2015;12: 1–15. doi: 10.1016/j.ecoser.2014.12.007

[23] Arkema KK, Verutes GM, Wood SA, Clarke-Samuels C, Rosado S, Canto M, et al Embedding ecosystem services in coastal planning leads to better outcomes for people and nature. Proc Natl Acad Sci. 2015;112: 7390–7395. doi: 10.1073/pnas.1406483112 26082545 10.1073/pnas.1406483112 PMC4475972

[24] Guerry AD, Polasky S, Lubchenco J, Chaplin-Kramer R, Daily GC, Griffin R, et al Natural capital and ecosystem services informing decisions: From promise to practice. Proc Natl Acad Sci. 2015;112: 7348–7355. doi: 10.1073/pnas.1503751112 26082539 10.1073/pnas.1503751112 PMC4475956

[25] Guerry A, Tallis H. Marine ecosystem services: A framework and practical set of tools for Ecosystem-Based Management In: Fogarty MJ, McCarthy J, editors. Marine Ecosystem-Based Management. Cambridge, MA: Harvard University Press; 2014 pp. 217–276.

[26] Ehler CN. A guide to evaluationg marine spatial plans. Paris: UNESCO Intergovernmental Oceanographic Commission; 2014. Report No.: 70.

[27] Rice J, Kidd S, Smith A. Marine spatial planning Marine Ecosystem-Based Management. Cambridge, MA: Harvard University Press; 2014.

[28] Apitz SE, Elliott M, Fountain M, Galloway TS. European environmental management: Moving to an ecosystem approach. Integr Environ Assess Manag. 2006;2: 80–85. doi: 10.1002/ieam.5630020114 16640322

[29] Gold B, Pastoors M, Babb-Brott D, Ehler C, King M, Maes F, et al. CALAMAR expert paper: integrated marine policies and tools working group. 2011; 23.

[30] Day J. The need and practice of monitoring, evaluating and adapting marine planning and management—lessons from the Great Barrier Reef. Mar Policy. 2008;32: 823–831. doi: 10.1016/j.marpol.2008.03.023

[31] Olsson P, Folke C, Hughes TP. Navigating the transition to ecosystem-based management of the Great Barrier Reef, Australia. Proc Natl Acad Sci. 2008;105: 9489–9494. doi: 10.1073/pnas.0706905105 18621698 10.1073/pnas.0706905105 PMC2474521

[32] Clarke C, Canto M, Rosado S. Belize Integrated Coastal Zone Management Plan. Coastal Zone Management Authority and Institute (CZMAI); 2013.

[33] Executive Order 13547—Stewardship of the Ocean, Our Coasts, and the Great Lakes [Internet]. Executive Order Jul 19, 2010. Available: https://obamawhitehouse.archives.gov/the-press-office/executive-order-stewardship-ocean-our-coasts-and-great-lakes

[34] Northeast Ocean Plan [Internet]. Northeast Regional Planning Body; 2016 Oct. Available: http://neoceanplanning.org/plan/

[35] Mid-Atlantic Regional Ocean Action Plan [Internet]. Mid-Atlantic Regional Planning Body; 2016 Nov. Available: boem.gov/Ocean-Action-Plan/

[36] Chin A, Kyne PM, Walker TI, McAULEY RB. An integrated risk assessment for climate change: analysing the vulnerability of sharks and rays on Australia’s Great Barrier Reef. Glob Change Biol. 2010;16: 1936–1953. doi: 10.1111/j.1365-2486.2009.02128.x

[37] Hobday AJ, Smith ADM, Stobutzki IC, Bulman C, Daley R, Dambacher JM, et al Ecological risk assessment for the effects of fishing. Fish Res. 2011;108: 372–384. doi: 10.1016/j.fishres.2011.01.013

[38] Williams A, Dowdney J, Smith ADM, Hobday AJ, Fuller M. Evaluating impacts of fishing on benthic habitats: A risk assessment framework applied to Australian fisheries. Fish Res. 2011;112: 154–167. doi: 10.1016/j.fishres.2011.01.028

[39] Samhouri JF, Levin PS. Linking land- and sea-based activities to risk in coastal ecosystems. Biol Conserv. 2012;145: 118–129. doi: 10.1016/j.biocon.2011.10.021

[40] Gaichas SK, Link JS, Hare JA. A risk-based approach to evaluating northeast US fish community vulnerability to climate change. ICES J Mar Sci J Cons. 2014;71: 2323–2342. doi: 10.1093/icesjms/fsu048

[41] Micheli F, De Leo G, Butner C, Martone RG, Shester G. A risk-based framework for assessing the cumulative impact of multiple fisheries. Biol Conserv. 2014;176: 224–235. doi: 10.1016/j.biocon.2014.05.031

[42] Stelzenmüller V, Fock HO, Gimpel A, Rambo H, Diekmann R, Probst WN, et al Quantitative environmental risk assessments in the context of marine spatial management: current approaches and some perspectives. ICES J Mar Sci J Cons. 2015;72: 1022–1042. doi: 10.1093/icesjms/fsu206

[43] Hare JA, Morrison WE, Nelson MW, Stachura MM, Teeters EJ, Griffis RB, et al A Vulnerability Assessment of Fish and Invertebrates to Climate Change on the Northeast U.S. Continental Shelf. PLOS ONE. 2016;11: e0146756 doi: 10.1371/journal.pone.0146756 26839967 10.1371/journal.pone.0146756 PMC4739546

[44] Levin PS, Fogarty MJ, Murawski SA, Fluharty D. Integrated Ecosystem Assessments: Developing the Scientific Basis for Ecosystem-Based Management of the Ocean. PLOS Biol. 2009;7: e1000014 doi: 10.1371/journal.pbio.1000014 19166267 10.1371/journal.pbio.1000014 PMC2628402

[45] Levin PS, Kelble CR, Shuford RL, Ainsworth C, deReynier Y, Dunsmore R, et al Guidance for implementation of integrated ecosystem assessments: a US perspective. ICES J Mar Sci. 2014;71: 1198–1204. doi: 10.1093/icesjms/fst112

[46] Colburn LL, Jepson M, Weng C, Seara T, Weiss J, Hare JA. Indicators of climate change and social vulnerability in fishing dependent communities along the Eastern and Gulf Coasts of the United States. Mar Policy. 2016; doi: 10.1016/j.marpol.2016.04.030

[47] Kappel CV, Halpern BS, Napoli N. Mapping cumulative impacts of human activities on marine ecosystems. Boston, MA: SeaPlan; 2012. Report No.: 03.

[48] Kildow JT, Colgan CS, Johnston P, Scorse JD, Farnum MG. State of the U.S. Ocean and Coastal Economies: 2016 Update. National Ocean Economics Program; 2016.

[49] Olsen SB, McCann JH, Fugate G. The State of Rhode Island’s pioneering marine spatial plan. Mar Policy. 2014;45: 26–38. doi: 10.1016/j.marpol.2013.11.003

[50] Zaykoski P, Shmookler R, Singer-Leavitt Z, Moura S, Gearon MS. Mid-Atlantic Regional Human Use Data Synthesis (HUDS) Project [Internet]. RPS ASA & SeaPlan; 2016. Available: https://www.openchannels.org/literature/13649

[51] Arkema KK, Verutes G, Bernhardt JR, Clarke C, Rosado S, Maritza Canto, et al Assessing habitat risk from human activities to inform coastal and marine spatial planning: a demonstration in Belize. Environ Res Lett. 2014;9: 114016 doi: 10.1088/1748-9326/9/11/114016

[52] Sharp R, Tallis H, Ricketts T, Guerry A, Wood SA, Chaplin-Kramer R, et al. InVEST [Internet]. The Natural Capital Project, Stanford University, University of Minnesota, The Nature Conservancy, and World Wildlife Fund; 2016. Available: http://www.naturalcapitalproject.org/software/

[53] Chung MG, Kang H, Choi S-U. Assessment of Coastal Ecosystem Services for Conservation Strategies in South Korea. PLOS ONE. 2015;10: e0133856 doi: 10.1371/journal.pone.0133856 26221950 10.1371/journal.pone.0133856 PMC4519238

[54] Duggan JM, Eichelberger BA, Ma S, Lawler JJ, Ziv G. Informing management of rare species with an approach combining scenario modeling and spatially explicit risk assessment. Ecosyst Health Sustain. 2015;1: 1–18. doi: 10.1890/EHS14-0009.1

[55] Ma S, Duggan JM, Eichelberger BA, McNally BW, Foster JR, Pepi E, et al Valuation of ecosystem services to inform management of multiple-use landscapes. Ecosyst Serv. 2016;19: 6–18. doi: 10.1016/j.ecoser.2016.03.005

[56] Patrick WS, Spencer P, Link J, Cope J, Field J, Kobayashi D, et al Using productivity and susceptibility indices to assess the vulnerability of united states fish stocks to overfishing. Fish Bull. 2010;108: 305–322.

[57] Kappel CV, Halpern BS, Selkoe KA, Cooke RM. Eliciting Expert Knowledge of Ecosystem Vulnerability to Human Stressors to Support Comprehensive Ocean Management In: Perera AH, Drew CA, Johnson CJ, editors. Expert Knowledge and Its Application in Landscape Ecology. Springer New York; 2012 pp. 253–277. doi: 10.1007/978-1-4614-1034-8_13

[58] Teck SJ, Halpern BS, Kappel CV, Micheli F, Selkoe KA, Crain CM, et al Using expert judgment to estimate marine ecosystem vulnerability in the California Current. Ecol Appl Publ Ecol Soc Am. 2010;20: 1402–1416. 10.1890/09-1173.1 20666257

[59] Moore RB, Johnston CM, Smith RA, Milstead B. Source and Delivery of Nutrients to Receiving Waters in the Northeastern and Mid-Atlantic Regions of the United States1. JAWRA J Am Water Resour Assoc. 2011;47: 965–990. doi: 10.1111/j.1752-1688.2011.00582.x 22457578 10.1111/j.1752-1688.2011.00582.x PMC3307627

[60] Admin 1—States, Provinces [Internet]. Natural Earth; Available: http://www.naturalearthdata.com/downloads/10m-cultural-vectors/

[61] Curtice C, Cleary J, Halpin P. Mid-Atlantic marine life data analysis team (MDAT) Final Report to MARCO. Duke Nicolas School of the Environment, Marine Geospatial Ecology Lab;

[62] Miththapala S. Tidal Flats [Internet]. Sri Lanka: IUNC; 2013. Report No.: 5. Available: https://cmsdata.iucn.org/downloads/tidal_flats.pdf

[63] How people benefit from New Hampshire’s Great Bay estuary A collaborative assessment of the value of ecosystem services and how our decision might affect those values in the future. [Internet]. NOAA Office for Coastal Management, New Hampshire Department of Environmental Services Coastal Program, and Eastern Research Group, Inc.; 2016 Available: https://www.des.nh.gov/organization/divisions/water/wmb/coastal/documents/greatbayesa-final-report-201611.pdf

[64] Pinsky ML, Worm B, Fogarty MJ, Sarmiento J, Levin SA. Marine taxa track local climate velocities. Science. 2013;341: 1239–1242. doi: 10.1126/science.1239352 24031017 10.1126/science.1239352

[65] Crain CM, Kroeker K, Halpern BS. Interactive and cumulative effects of multiple human stressors in marine systems. Ecol Lett. 2008;11: 1304–1315. doi: 10.1111/j.1461-0248.2008.01253.x 19046359 10.1111/j.1461-0248.2008.01253.x

[66] Teichert N, Borja A, Chust G, Uriarte A, Lepage M. Restoring fish ecological quality in estuaries: Implication of interactive and cumulative effects among anthropogenic stressors. Sci Total Environ. 2016;542, Part A: 383–393. doi: 10.1016/j.scitotenv.2015.10.068 26520263 10.1016/j.scitotenv.2015.10.068

[67] Halpern BS, Frazier M, Potapenko J, Casey KS, Koenig K, Longo C, et al Spatial and temporal changes in cumulative human impacts on the world’s ocean. Nat Commun. 2015;6: 7615 doi: 10.1038/ncomms8615 26172980 10.1038/ncomms8615 PMC4510691

[68] Stock A, Micheli F. Effects of model assumptions and data quality on spatial cumulative human impact assessments. Glob Ecol Biogeogr. 2016;25: 1321–1332. doi: 10.1111/geb.12493

