# Supplementary material for: Habitat risk assessment for regional ocean planning in the U.S. Northeast and Mid-Atlantic
Source: PLoS One. 2017 Dec 20;12(12):e0188776. doi: 10.1371/journal.pone.0188776 (PMC5737885; doi:10.1371/journal.pone.0188776)
Supplement: S1 File — Additional explanation and details for the methods and data used in this study including estimating risk, habitat data, human activities and stressor data, and the wind energy analysis. Table A, Exposure and consequence criteria used in the Habitat Risk Assessment model; Fig A, Habitat types; Table B, Habitat data and sources; Table C, Human activity and stressor data; Fig B, Spatial distribution of aquaculture of five kinds of fishing; Fig C, Human structures; Fig D, Human trampling, shipping, and shipwrecks; Fig E, Military danger zones, ocean dumping, sand and gravel mining, and oil spills; Fig F, Tourist activities; Fig G, Land-based stressors; Fig H, Increasing sea surface temperature. (DOCX) [file pone.0188776.s001.docx]

# Supporting Information (S1 file)

## Risk Assessment Criteria

We use the criteria developed through the Massachusetts cumulative impact assessment (detailed in Kappel et al. [1]), which were based on expert elicitation, and modify them for use in the HRA exposure and consequence framework. This expert elicitation asked scientists to score, for each criteria included in the cumulative impact analysis (‘vulnerability criteria’) and for areas in which they had expertise, the effect of each stressor. From the cumulative impact assessment criteria, we use frequency of occurrence and scale of effect as exposure criteria in our analysis. In addition, we add an exposure criterion to capture intensity. For consequence criteria, we use change in biomass, trophic impact, and expected recovery time from the cumulative impacts criteria (Table A in S1). In total, we use seven criteria, including all five of the cumulative impacts criteria [1].

**S1. Table A. Exposure and consequence criteria used in the Habitat Risk Assessment model.**

|  | **Criteria** | **Source** | **Description** |
| --- | --- | --- | --- |
| Exposure | Spatial overlap | Spatial habitat and stressor data (see Table B & C in S1). | The overlap in space of a given habitat and stressor.* |
|  | Intensity | Spatial stressor data (see Table B in S1). | Where available, spatial variation in the intensity of the stressor (see Table B in S1). |
|  | Frequency of occurrence | Kappel et al. 2012 | Relative frequency that the stressor occurs at a given location (originally days per year in Kappel et al. 2012) |
|  | Spatial scale of effect | Kappel et al. 2012 | Relative spatial scale at which a single occurrence of the stressor impacts the ecosystem, both directly and indirectly (originally km^2^ in Kappel et al. 2012) |
| Consequence | Change in biomass | Kappel et al. 2012 | Relative change in biomass of the affected ecosystem component compared to its ‘natural’ state (originally percent in Kappel et al. 2012) |
|  | Trophic impact | Kappel et al. 2012 | Relative extent of marine life affected by a stressor within a given ecosystem (i.e. single or multiple species, single or multiple trophic levels, or the entire ecosystem) |
|  | Expected recovery time | Kappel et al. 2012 | Relative recovery time required for the ecosystem to return to natural conditions (originally in years in Kappel et al. 2012) |

*Note that this criterion, ‘spatial overlap’, is used differently from other criteria: if habitat and stressor overlap, then the model calculates exposure and consequence scores using the criteria in the table. If a habitat and stressor do not overlap, then risk from that stressor to that habitat=0.

We scale and bin each continuous cumulative impacts vulnerability criteria score for use in the HRA model (see Table A in S1 for the original units of each criteria). To do this, we normalize the range of cumulative impact criteria scores by finding the maximum score and then assigning scores less than one third of the maximum score the lowest rank, 1; scores between 1/3 and 2/3 of the of the maximum score a rank of 2; and scores greater than 2/3 of the maximum score the maximum rank of 3. This approach formats the scores for HRA (which requires ranked 1-3 scoring) and improves interpretability, while preserving the relative rank of scores and the relative importance of high scores.

We did not further weight scores of exposure and consequence in our analysis. Cumulative impacts analyses use methods from decision theory to weight each criterion based on its perceived importance relative to other criterion. We do not use this weighting because the HRA equation effectively weights measures of exposure and consequence within the analysis (equations 1-3, main text) and we worried that additional weighting would reduce transparency and interpretability. Additionally, the normalization of vulnerability scores described above accomplishes one of the reasons cumulative impacts analysis uses these weights, which is to transform the alternative scales of vulnerability scores (e.g., km^2^ and occurrences/year) into a single, comparable scale.

## Habitats

To quantify and map risk to habitats, we first classify the Atlantic coastal area—from Maine to Virginia—into 13 habitat types, ranging across nearshore and offshore zones (Fig A in S1, coastline adapted from Natural Earth [2]). To build on and to allow for comparisons with previous habitat risk assessment work in the region [1], we use the classification scheme used in the cumulative impact assessment of Massachusetts waters. The use of this scheme also ensures that we appropriately apply the expert-elicited scores to the habitats they were determined for. Across habitats, we select the most recent data that are available nationally or have already been synthesized regionally (Table B in S1).


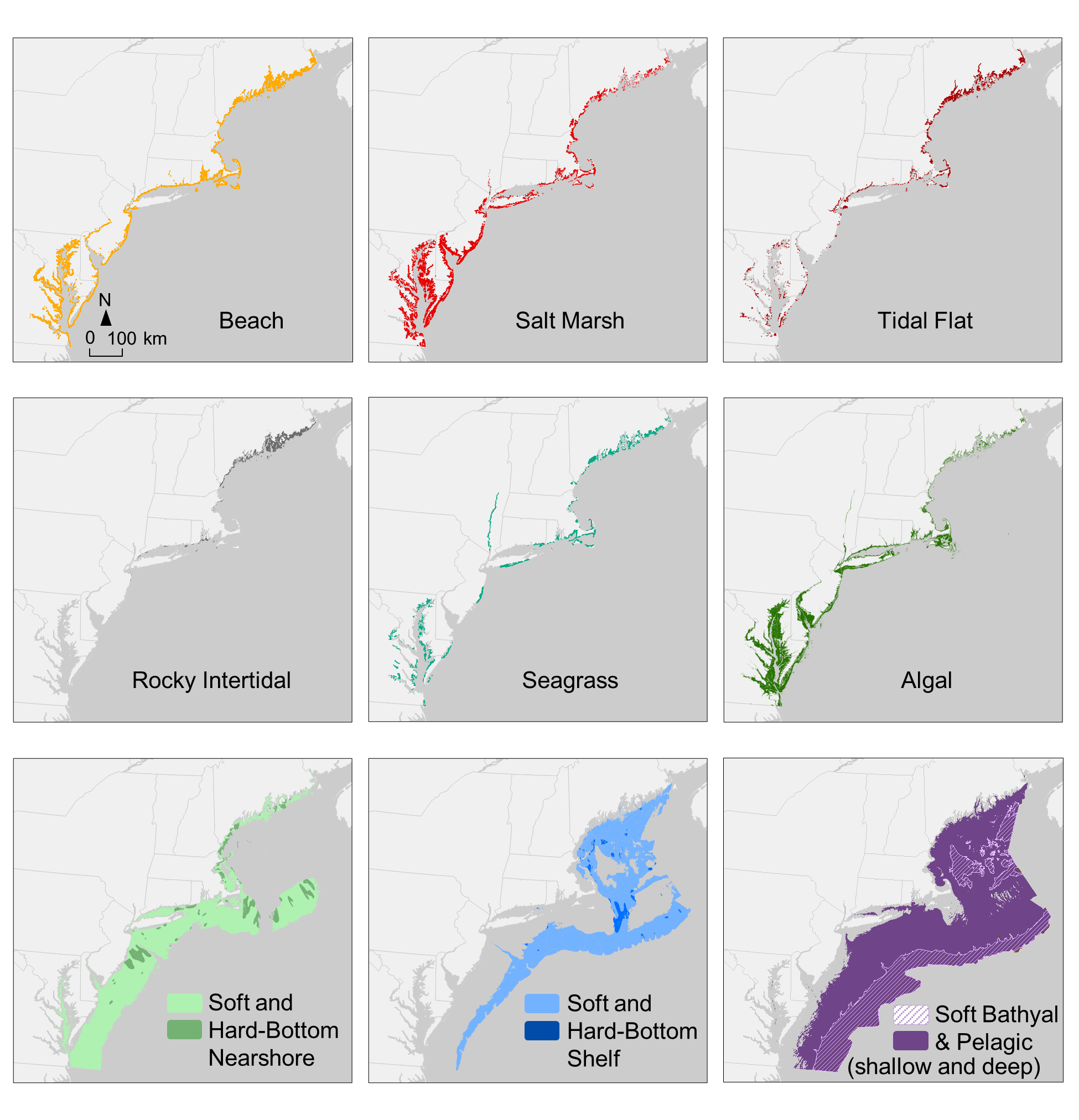
**S1. Fig A. Habitat types.** Note that shallow and deep pelagic habitats overlap in two-dimensional space, as visualized, but not in three-dimensional space.

**S1. Table B. Habitat data and sources.**

| **Habitat** | **Source** | **Year** | **Description** |
| --- | --- | --- | --- |
| **Nearshore** | | | |
| **Beach** | NOAA Office of Response and Restoration, Environmental Sensitivity Maps [3] | 2001-2014, depending on the state | Polyline data (for all states except ME) for fine-to-medium grained, coarse grained sand, and mixed-grain sand beaches; sand scarps; and steep slopes (ESI codes 3*,4*, & 5*). Polyline data buffered by 50m. |
| **Tidal flats** | NOAA Office of Response and Restoration, Environmental Sensitivity Maps [3] | 2001-2014, depending on the state | Polygon data for exposed (ESI code 7*) and sheltered (ESI code 9A) tidal flats. |
| **Rocky intertidal** | US Fish & Wildlife National Wetlands Inventory [4] | 2016 | Marine (NWI code M2RS*) and estuarine (NWI code E2RS*) intertidal rocky (bedrock or rubble) shores |
| **Salt marsh** | US Fish & Wildlife National Wetlands Inventory [4] | 2016 | Estuarine, intertidal, and emergent and can be persistent, non-persistent, and are often characterized by *phragmites australis* (NWI E2EM*) |
| **Seagrass** | Northeast Ocean Data for Eelgrass beds [5]; NOAA “Seagrasses in the continental United States as of March 2015” [6] | 2014; 2015 | Aquatic vascular vegetation beds dominated by submerged, rooted, vascular species or submerged or rooted floating freshwater tidal vascular vegetation. Updated NEOD with NOAA data for full spatial coverage. |
| **Algal zone** | NOAA National Centers for Environmental Information US Coastal Relief Model^1^ [7] | 1999 | Nearshore, subtidal habitat (<10m) not otherwise occupied by a nearshore habitat. |
| **Nearshore soft-bottom** | NOAA National Centers for Environmental Information US Coastal Relief Model^1^ [7]; and  U.S. Geological Survey Continental Margin Mapping [8] | 1999, 2005 | Subtidal habitat 10-60m in depth with mud, sil, and sand sediment |
| **Nearshore hard-bottom** | NOAA National Centers for Environmental Information US Coastal Relief Model^1^ [7]; and  U.S. Geological Survey Continental Margin Mapping [8] | 1999, 2005 | Subtidal habitat 10-60m in depth with cobble, boulder, or bedrock sediment |
| **Offshore** | | | |
| **Soft-bottom shelf** | NOAA National Centers for Environmental Information US Coastal Relief Model^1^ [7]; and  U.S. Geological Survey Continental Margin Mapping [8] | 1999, 2005 | Subtidal habitat 60-200m in depth with mud, sil, and sand sediment |
| **Hard-bottom shelf** | NOAA National Centers for Environmental Information US Coastal Relief Model^1^ [7]; and  U.S. Geological Survey Continental Margin Mapping [8] | 1999, 2005 | Subtidal habitat 60-200m in depth with cobble, boulder, or bedrock sediment |
| **Bathyal shelf** | NOAA National Centers for Environmental Information US Coastal Relief Model^1^ [7]; and  U.S. Geological Survey Continental Margin Mapping [8] | 1999, 2005 | Subtidal habitat > 200m in depth |
| **Shallow pelagic** | NOAA National Centers for Environmental Information US Coastal Relief Model^1^ [7] | 1999, 2005 | The water column above 200m in areas > 30m deep |
| **Deep pelagic** | NOAA National Centers for Environmental Information US Coastal Relief Model^1^ [7]; and  U.S. Geological Survey Continental Margin Mapping [8] | 1999, 2005 | The water column below 200m |

^1^ The NOAA Coastal Relief Model was the only raster data input for habitat classifications. Its resolution is 1-arc second.

#### Nearshore

Beach and tidal flat habitats are derived from NOAA’s Environmental Sensitivity Index (ESI) maps [3], which catalog coastal shorelines and resources. To define beaches for this analysis we subset the ESI polyline categories to select for fine to medium-grained sand beaches, coarse-grained sand beaches, mixed-grain sand beaches, and sand scarps and steep slopes. With the exception of Maine, which includes polygon beach data, these data are represented by ESI lines, which we buffer by 50m (Table B in S1). Tidal flats are defined by (and subset for) ESI polygons of exposed tidal flats and hypersaline tidal flats. States update their ESI data at variable times: ESI data from Delaware, New Jersey, and Pennsylvania are from 2014; Maine and Maryland from 2007; Virginia from 2005; New Hampshire from 2004; and Massachusetts, Rhode Island, Connecticut, and New York from 2001 (Table B in S1). These represent some of the older data within the analysis, but are the latest, reliable data available for this stressor. We merge statewide data, subset for each habitat classification, in ArcGIS to create spatially continuous coverage across the Atlantic for each habitat.

Rocky intertidal and salt marsh habitats are derived from U.S. Fish and Wildlife (USFW) National Wetlands Inventory (NWI) [4]. NWI organizes coastal geospatial data by state and watershed to map and project trends in national wetlands. Data were last updated in 2016. Rocky intertidal habitats are defined as estuarine or marine intertidal rocky shores, which consist of bedrock or rubble, and we subset the corresponding codes from the complete NWI dataset (Table B in S1). Salt marshes are defined as estuarine, intertidal, and emergent and can be persistent, non-persistent, and are often characterized by *phragmites australis;* we again subset the corresponding codes from the complete dataset. We merge statewide data, subset for each habitat classification, in ArcGIS to create spatially continuous data across the Atlantic for each habitat (Table B in S1).

To map seagrass, we combine datasets from multiple sources. We use eelgrass data from the Northeast Ocean Data (NEOD) portal [5], which compiles state-based datasets from 2014 from Connecticut, Rhode Island, Massachusetts, New Hampshire, and Maine. In the Mid-Atlantic, we use 2015 NOAA seagrass data from the federal Marine Cadastre (Table B in S1) [6]. To create spatially continuous data, we merge the two data sources in ArcGIS, prioritizing the more accurate statewide data were it exists. Together, these data cover the entirety of our area of analysis.

Algal habitats are defined as those areas shallower than 10m in depth not otherwise occupied by a nearshore habitat (Table B in S1). This follows the protocol outlined in Kappel et al. [1]. We select depths less than 10m using the ArcGIS raster calculator from the U.S. Coastal Relief Model created by NOAA’s National Centers for Environmental Information (NCEI, 1999) [7]. We then convert this raster to vector, polygon format. Though this is the oldest dataset we use, we do not expect that bathymetry data have changed much in the proceeding 20 years and NCEI represent the most complete and accurate dataset available. Data extend to the outer reaches of the continental shelf. In ArcGIS, we merge the nearshore habitats (beaches, tidal flat, rocky intertidal, salt marsh, and seagrass) described above and then erase these areas from the depth based data described, resulting in areas shallower than 10m not otherwise classified by another habitat.

Nearshore hard- and soft-bottom habitats are those areas deeper than 10m and shallower than 60m (Table B in S1). We use the ArcGIS raster calculator to select this depth range from the NCEI bathymetry data described above [7] and then convert this subset to vector format. Sediment data are from U.S. Geological Survey’s (USGS) ‘Continental Margin Mapping (CONMAP) sediments grain size distribution for the Unites States East Coast Continental Margin (2005)[8], which are the most accurate and spatially complete data available. The CONMAP data have full spatial coverage across our area of analysis. Soft-bottom habitats are those with mud, silt, and sand. Hard-bottom habitats were those with cobble, boulders, and bedrock. We intersect the depth data with the sediment data to define the area of nearshore soft- and hard-bottom habitat.

#### Offshore

We define offshore habitats by their depth and sediment type following the delineations outlined in the work by Kappel et al. [1]. Depth data are from the NCEI bathymetry data [7] and sediment data are from CONMAP (both sources described above) [8]. Shelf habitat are selected as areas from 60-200m in depth, again using the NCEI bathymetry data. For shelf habitat, soft-bottom classification includes areas with mud, silt, and sand, while hard-bottom classifications are those with cobble, boulders, and bedrock; we intersect sediment selections with depth selections to define the spatial extent of soft- and hard-bottom shelf. Bathyal shelf habitat is defined and selected as areas greater than 200m in depth, regardless of sediment. In waters deeper than 30m, we divide the water column into shallow (< 200m deep) and deep (> 200m deep) pelagic zones, again regardless of sediment type (Table B in S1). Note that some of these habitats overlap in 2D space, but not in 3D space (Fig A in S1).

## Human Activities and Stressors

To assess the cumulative risk of human activities to coastal and marine habitats in the region, we incorporate the impact of 31 stressors. Using Kappel et al.’s [1] analysis as a starting point, we prioritize the inclusion of stressors based on engagement with the regional planning bodies, data availability across both regions, and the availability of expert-elicited vulnerability scores [9]. We use only those stressors for which there are vulnerability scores [1] and select data to match the human activities they were derived for. We attempt to represent each included stressor as completely as possible, but in some cases are limited by the extent of available data (Table C in S1). We group these 31 stressors into themes consisting of fishing and aquaculture, human structures, coastal uses and impacts, land-based impact, and climate change.

**S1. Table C. Human activity and stressor data.**

| **Stressor** | **Source** | **Year** | **Resolution** | **Coverage** | **Description** | **Intensity** |
| --- | --- | --- | --- | --- | --- | --- |
| **Fishing and Aquaculture** | | | | | | |
| **Fishing—recreational** | Northeast Ocean Data Portal ‘recreational fishing effort’ [10] | 2000-2009 | 500m | Northeast and Mid-Atlantic | Number of trips from 2000-2009 based on vessel trip report cards. Federally permitted party boats and charter boats. | Variable based on effort |
| **Fishing—artisanal non-destructive** | NOAA Northeast Fishery Science Center | 2006-2015 avg. | 500m | Northeast and Mid-Atlantic | Cast net, hand line or rod and reel, harpoon | Variable based on effort |
| **Fishing—demersal destructive** | NOAA Northeast Fishery Science Center | 2010-2015 avg. | 500m | Northeast and Mid-Atlantic | Dredge: ocean quahog/surf clam, mussel, sea-scallop, scallop chain mat, urchin, and other; otter trawl: haddock separator, beam, bottom scallop, bottom fish, ruhle, bottom shrimp, bottom twin, bottom other; bottom pair trawl; hand rake | Variable based on effort |
| **Fishing—non-destructive demersal** | NOAA Northeast Fishery Science Center | 2010-2015 avg. | 500m | Northeast and Mid-Atlantic | Diving gear; bottom longline; pot: crab, eel, fish, hag, lobster, shrimp, conch/whelk, mixed, other; trap | Variable based on effort |
| **Fishing—pelagic** | NOAA Northeast Fishery Science Center | 2006-2015 avg. | 500m | Northeast and Mid-Atlantic | Pelagic long line; Fyke net; gill net: large mesh drift, runaround, sink, small mesh drift, other; midwater otter trawl; midwater pair trawl; seine: purse, Danish, haul, Scottish, stop; weir | Variable based on effort |
| **Aquaculture—finfish** | Northeast Ocean Data of Human Dimensions, Aquaculture [11] | 2014 | Vector | Northeast | Atlantic cod, Atlantic salmon, and Steelhead trout | Binary |
| **Aquaculture—shellfish** | Northeast Ocean Data of Human Dimensions, Aquaculture [11] | 2014 | Vector | Northeast | blue mussel, hard and soft clam, oyster, scallop, quahog, and combinations thereof | Binary |
| **Aquaculture—marine plants** | Northeast Ocean Data of Human Dimensions, Aquaculture [11] | 2014 | Vector | Northeast | Seaweed | Binary |
| **Human Structures** | | | | | | |
| **Benthic structures** | Northeast Ocean Data of Human Dimensions “cable and pipeline areas” [12] | 2011-2012 | Vector | Atlantic | Known cable and pipelines | Binary |
| **Energy infrastructure—liquid natural gas** | U.S. Energy Information Administration “Liquefied Natural Gas Import/Export terminals” [13] | 2013 | Vector | National | LNG import and export terminals, buffered by 3km | Binary |
| **Energy infrastructure—tidal** | Northeast Ocean Data of Human Dimensions “Marine Hydrokinetic Projects” [12] | 2016 | Vector | Atlantic | Tidal and wave energy projects as submitted to the Federal Energy Regulatory Commission, buffered by 6km | Binary |
| **Energy infrastructure—wind** | Northeast Ocean Data of Human Dimensions “Block Island Turbine locations” [12] | 2015 | Vector | Northeast and Mid-Atlantic | 5 individual wind turbines, buffered by 1030m | Binary |
| **Coastal engineering** | NOAA Office of Response and Restoration, Environmental Sensitivity Maps [3] | 2001-2014, by state | Vector | National | Exposed and sheltered man-made structures (solid or riprap) (ESI codes 1B*, 6B*, 8C*). Polyline data (for all states except ME) buffered by 250m. Hardened shorelines can modify or destroy habitat through direct impact and through altered circulation and sediment transport. | Binary |
| **Additional Coastal Uses and Impact** | | | | | | |
| **Human trampling** | Oak Ridge National Laboratory’s LandScan data [14] | 2012 | 1km | National | Human population as a proxy for human trampling by foot, buffered by 250m | Variable |
| **Dredging** | Mid-Atlantic Ocean Data Portal “maintained channels” [15] | 2015 | Vector | National | Coastal channels maintained by the U.S. Army Corps of Engineers | Binary |
| **Shipping** | Northeast Ocean Data from Automatic Identification System (AIS) vessel tracking “Marine Transportation” [16] | 2012 | 100m | National | All boat types with AIS tracking (>30m). This impact accounts for noise and light pollution as well as direct impacts. | Variable based on density |
| **Shipwrecks** | NOAA National Ocean Service “wrecks and obstructions” [17] | 2011 | Vector | National | All wrecks (pt. data), with a 50m buffer | Binary |
| **Military activity** | National Marine Cadastre “danger zones and restricted areas” [18] | 2016 | Vector | National | Areas used by the military (danger zones) and those that are “restricted areas” due to government use | Binary |
| **Ocean dumping** | National Marine Cadastre “ocean disposal sites” [19] | 2016 | Vector | National | Locations where permitted ocean dumping is permitted | Binary |
| **Ocean mining** | National Marine Cadastre “Federal [outer continental shelf] OCS sand and gravel borrow (lease areas)” [20] | 2015 | Vector | National | Active federal sand and gravel borrow areas | Binary |
| **Oil spills (ocean pollution)** | U.S. Coast Guard “marine casualty and pollution database” [21] | 2002-2015 | Vector | National | Oil spills > 10 gallons, with extent estimated by total volume spread to a thickness of 0.001mm | Binary |
| **Tourism—kayaking** | Northeast Ocean Data “board and paddle events” [10],  Mid-Atlantic Ocean Data Portal statewide recreational uses workshops (for NY, NJ, DE, MD, and VA) [22] | 2015, 2012 | Vector | Northeast and Mid-Atlantic | Board and paddle events (buffered by 3km) and dominant areas for recreational kayaking, non-motorized vessels, and board and rowing. | Binary |
| **Tourism—recreational boating** | Northeast Ocean Data “recreational boater route density” [10],  Mid-Atlantic Ocean Data Portal statewide recreational uses workshops (for NY, NJ, DE, MD, and VA) [22] | 2012 | 250m | Northeast and Mid-Atlantic | Upper quartile density of boater density merged with dominant areas for charter and personal recreational boating | Binary |
| **Tourism—SCUBA** | Northeast Ocean Data “recreational SCUBA diving areas” [10],  Mid-Atlantic Ocean Data Portal statewide recreational uses workshops (for NY, NJ, DE, MD, and VA) [22] | 2015, 2012 | Vector | Northeast and Mid-Atlantic | Recreational SCUBA areas and dominant areas for charter diving and snorkeling, recreational dive fishing, and scuba and snorkeling diving | Binary |
| **Tourism—surfing** | Northeast Ocean Data “board and paddle events” [10],  Mid-Atlantic Ocean Data Portal statewide recreational uses workshops (for NY, NJ, DE, MD, and VA) [22] | 2015, 2012 | Vector | Northeast and Mid-Atlantic | SUP and surf races and contests (buffered by 3km), and dominant paddling and surface water sports areas. | Binary |
| **Tourism—wildlife viewing** | Northeast Ocean Data “commercial whale watching” [10],  Mid-Atlantic Ocean Data Portal statewide recreational uses workshops (for NY, NJ, DE, MD, and VA) [22] | 2015, 2012 | Vector | Northeast and Mid-Atlantic | Commercial areas for whale watching and dominant areas for charter, offshore, and recreational wildlife viewing and charter scenic viewing. | Binary |
| **Land-Based Impact** | | | | | | |
| **Power plants** | Environmental Protection Agency “coastal energy facilities” [23] | 2012 | Vector | Eastern U.S. | Coastal energy facilities that generate electricity, buffered by 3km. Represents the entrainment of larvae and small plants from intake piped used for cooling | Binary |
| **Light pollution** | Modeled using cumulative viewsheds from ArcGIS, based on NOAA National Centers for Environmental Information “nighttime lights” [24] | 2013 | 500m | Global | Light intensity modeled to 32km offshore using ArcGIS viewshed tool. | Variable, based on intensity |
| **Inorganic pollution** | National Land Cover Database;  USGS Hydrological units [25] | 2011, ND | Vector | National | Percentage of the watershed that’s impervious used as a proxy for non-point source pollution from cars, roads, and urban areas. | Variable |
| **Nutrient pollution** | USGS SPARROW model [26] | 2002 | Vector | National | Total nutrient loading adjusted by estuary volume to produce a relative indicator loading between estuaries. | Variable based on concentration |
| **Climate Change** | | | | | | |
| **Sea-surface temperatures (SST)** | NOAA’s International Comprehensive Ocean-Atmospheric Datasets [27] | 1960-2015 | 1 decimal degree | Global | Relative rate of rising SST across the entire area of interest | Variable based on relative rate of warming |

#### *Variable Intensity*

For many human activities and stressors outlined below, we add to the spatial representation of the data by including a spatially-explicit intensity rank (Table A in S1). This spatially-explicit intensity is included in the analysis as an exposure criterion factored into the risk equation (equation 1, main text). In the absence of spatially explicit intensity, we do not include intensity as a component of exposure in order to avoid assuming an intensity level where there are not data to support it. To determine intensity, we first define the quartiles of effort or use. For example, with shipping, these quartiles represent variation in the density of shipping traffic. We then give the area in the lowest quartile the lowest intensity ranking, areas falling in the middle two quartiles a moderate intensity ranking, and those areas in the top quartile the high intensity ranking. This approach allows us to include the full range of data for these activities, while also accounting for variable impacts on habitats. We apply this methodology, where data allow, for fishing effort, human trampling, shipping, and sea surface temperatures (Table C in S1).

#### Fishing and Aquaculture

We source aquaculture data for finfish predators, shellfish, and marine plants from the NEOD portal [11]. We do not have full spatial coverage for this stressor: at the time of our analysis, town-level permitted aquaculture in Massachusetts and data for the Mid-Atlantic were not available. From the aquaculture data available from the Portal we subset the data based on the ‘all species’ and ‘general species’ attributes: finfish aquaculture includes Atlantic cod, Atlantic salmon, and Steelhead trout; shellfish aquaculture includes shellfish, CT shellfish beds, blue mussel, hard and soft clam, oyster, scallop, quahog, and combinations thereof; aquaculture for marine plants consists entirely of seaweed (Fig B, Table C in S1).
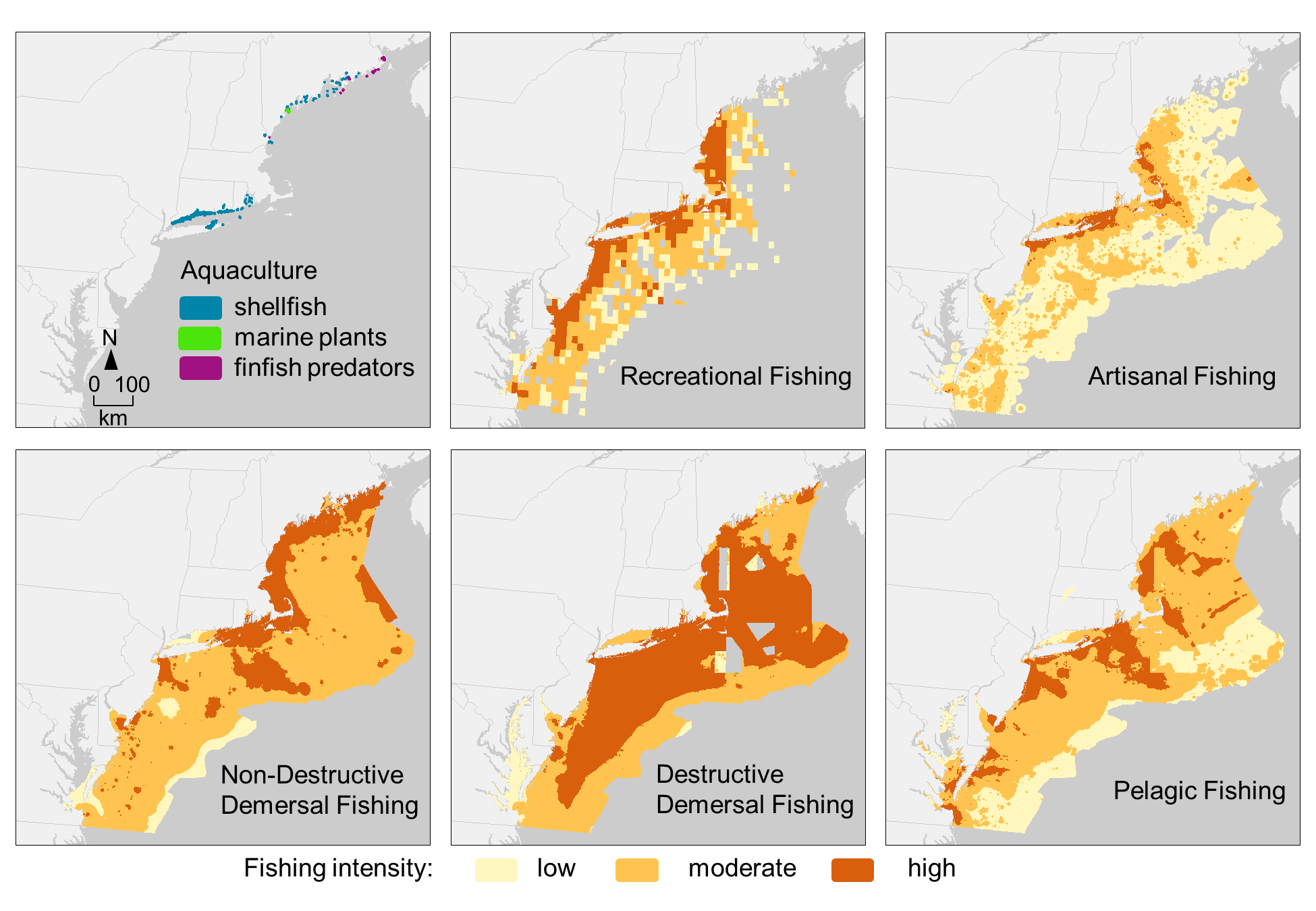
**S1. Fig B. Spatial distribution of aquaculture of five kinds of fishing.** Maps for fishing include three levels of intensity.

We source recreational fishing data from NEOD [10]. These data, compiled in 2011 by the Nature Conservancy from the National Marine Fisheries Service, summarize the number of recreational trips taken to 10-minute squares across the Atlantic from 2000-2009. The data include the relative effort from federally permitted party boats and charts boats (Table C in S1). While the data do not include individual recreational fishing trips, we assume that patterns of relative effort are consistent with these fishers. We scale intensity based on this variable effort using the methodology described above.

We source data on artisanal, destructive demersal, non-destructive demersal, and pelagic fishing data from NOAA’s Northeast Fisheries Science Center (NEFSC), which combines both Vessel Trip Report (VTR) and National Fisheries Observer Program data (Table C in S1). VTR data only ask for a single latitude/longitude point for each gear and statistical area fished, providing an imprecise measure of the spatial extent of fishing effort. Using Observer Program data for matched records that have information about the spatial extent of haul beginning and end points, DePiper [28] estimated cumulative distribution functions via duration models, estimating the effect of attributes that may influence haul extent, such as gear type and trip duration. These cumulative distribution functions spatially distribute a given trip’s effort around their reported VTR latitude and longitude, providing much higher spatial resolution of effort and precision about a haul’s location (Fig B in S1). The maps provided by NEFSC present long run average effort in terms of days-at-sea for a range of gear types and across our entire area of analysis. We use quartiles of effort data to scale intensity for each fishing type as outlined above using the combined distribution of all fishing types to define the quartiles (Fig B in S1). Due to issues of confidentiality with VTR records, these data require summarizing over longer time intervals and coarser gear classifications than could be supported by the raw data. For demersal destructive and non-destructive categories, this required that we summarize data from 2010-2015 (Table B in S1). Because there are fewer records for artisanal and pelagic categories, we use data summarized across 2006-2015 for these categories (Table B in S1).

#### Human Structures

Additional stressors related to human structures include benthic structures, three types of energy-related structures, and coastal engineering. Benthic structures are comprised of known cable and pipeline areas as included in the NEOD portal of ocean uses [12], which has full spatial coverage and was not further manipulated for use (Fig C in S1). We include import and export LNG sites from the Energy Information Administration at a national scale [13] and buffer these point data by 3km following methodology in Kappel et al. [1]. We include approved tidal and hydrokinetic energy sites from the NEOD portal [12] and buffer these point data by 3km following methodology in Kappel et al. [1]. Both LNG and tidal related energy data are relatively recent (2013, 2016) and have full coverage across the Atlantic (Table C in S1). We map the five newly created wind turbines off of Block Island, Rhode Island [12] and buffer each point by 1050m, seven times the rotor blade diameter and the rule-of-thumb for spacing, to address localized turbulence and wake effects [29](Fig C, Table C in S1). Coastal engineering includes types of hardened shoreline, which can affect habitats through direct impact and by altering flow dynamics. To map coastal engineering, we use NOAA’s Environmental Sensitivity Index (ESI) data [3] for all relevant states to subset shorelines with exposed and sheltered man-made structures (solid or riprap) using ESI attribute ‘most sensitive’. We then apply the averaged vulnerability scores from Kappel et al. [1] two coastal engineering subcategories; we assume that coastal engineering impacts the adjacent 250m of coastal areas and buffer the polyline data by that amount (again, following Kappel et al. [1] methodology; Fig C, Table C in S1). All structural data were presence/absence and contained no variability in their intensity.
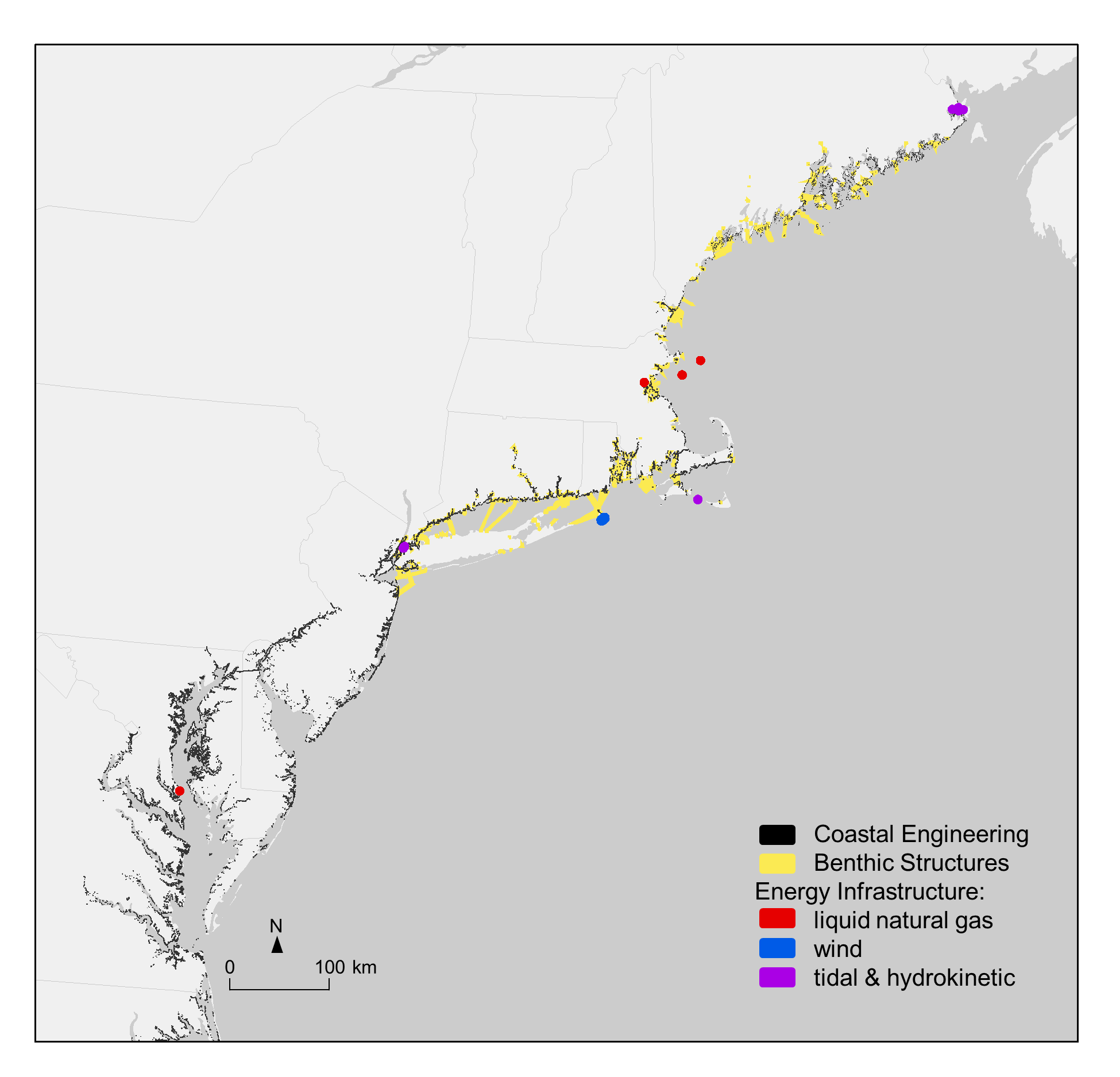


**S1. Fig C. Human structures.**

#### Additional coastal uses and impacts

We map thirteen components of coastal uses that have direct physical impacts on habitats: human trampling, dredging, shipping, shipwrecks, military activity, ocean dumping, ocean mining, oil spills, and five types of tourist activities (Figs D-F, Table C in S1).

We map human trampling, dredging, shipping, and shipwrecks using a variety of data sources (Table C in S1). Human trampling captures the use natural areas by humans on foot. As a proxy for this impact, we map estimated human population along the coast using Oak Ridge National Laboratory’s LandScan data (1-km resolution) of human population [14]. We scale the intensity, following the previously outlined methodology, of the human trampling based on population density within a 25km window, convert raster data to vector, and buffer by 250m to capture the impact of trampling on nearshore habitats (Fig D, Table C in S1). We use maintained channel data, which have recent coverage across the Atlantic from the Mid-Atlantic Regional Ocean Council (MARCO) data portal [15], to represent dredging without further manipulation (Table C in S1). We map shipping using the NEOD portal’s vessel activity [16], which is based on Automatic Identification System vessel tracking data, for all densities and boat types for 2012, the most recent year available at the time of analysis. We use average traffic density to define intensity following methodology above and convert to vector format for use in the model (Fig D, Table C in S1). To capture shipwrecks, we use data from the most recent (2011) ‘wrecks and obstructions’ data [17] available on the national Marine Cadastre and buffer these point data by 50m to convert to polygon for use in the model (Fig D, Table C in S1).

**
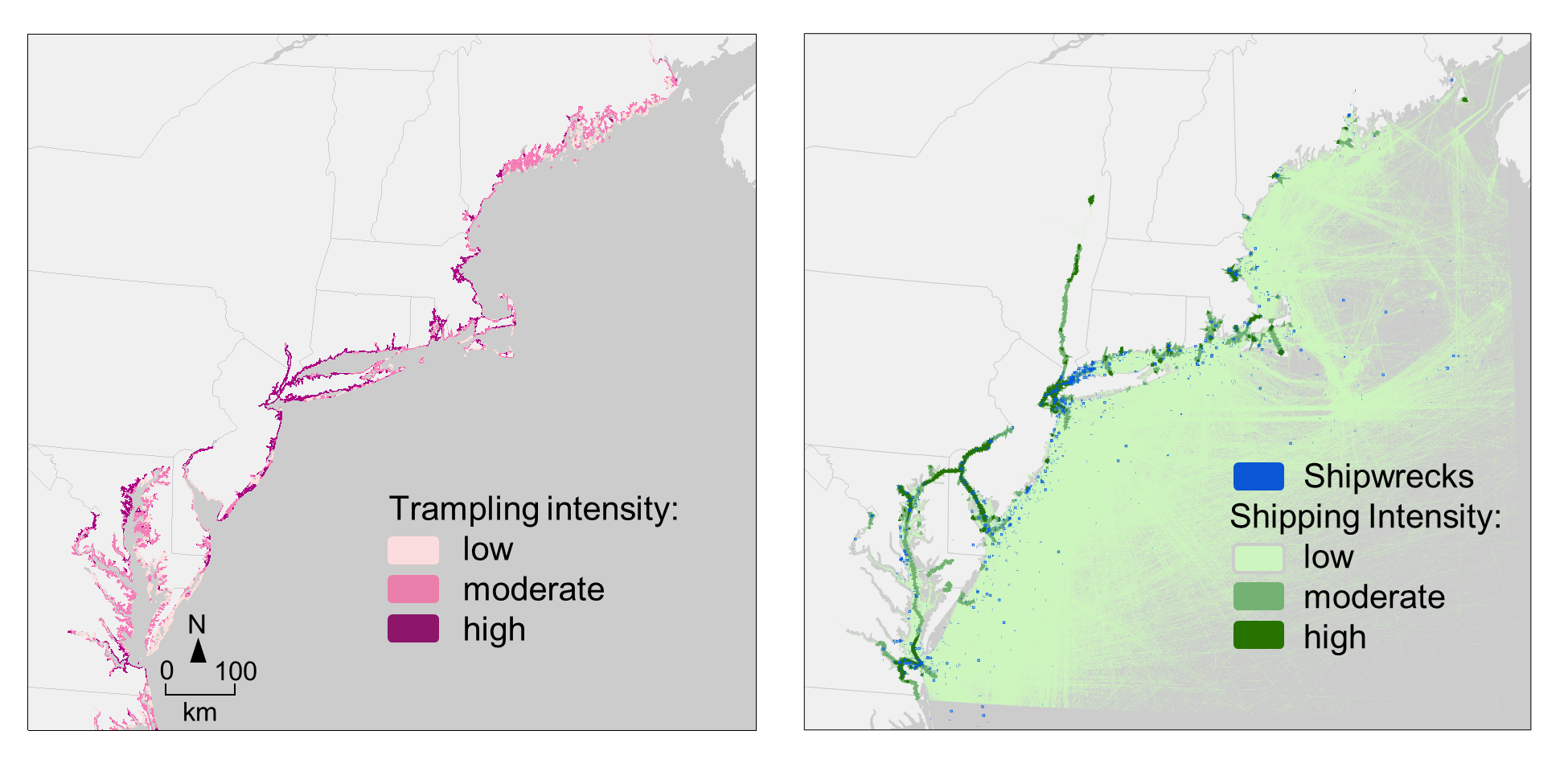
S1. Fig D. Human trampling (left), shipping, and shipwrecks (right).** Human trampling and shipping include three levels of intensity.

We map military activity based on Danger Zones and Restricted Areas for 2016 as outlined by the Code of Federal Regulations, provided by NOAA’s Office for Coastal Management, and available on the National Marine Cadastre (Fig E in S1) [18]. A danger zone is defined as, "A defined water area (or areas) used for target practice, bombing, rocket firing or other especially hazardous operations, normally for the armed forces.” A restricted area is defined as, "A defined water area for the purpose of prohibiting or limiting public access to the area. Restricted areas generally provide security for Government property and/or protection to the public from the risks of damage or injury arising from the Government's use of that area.” We use these spatial data without further manipulation (Table C in S1).

**
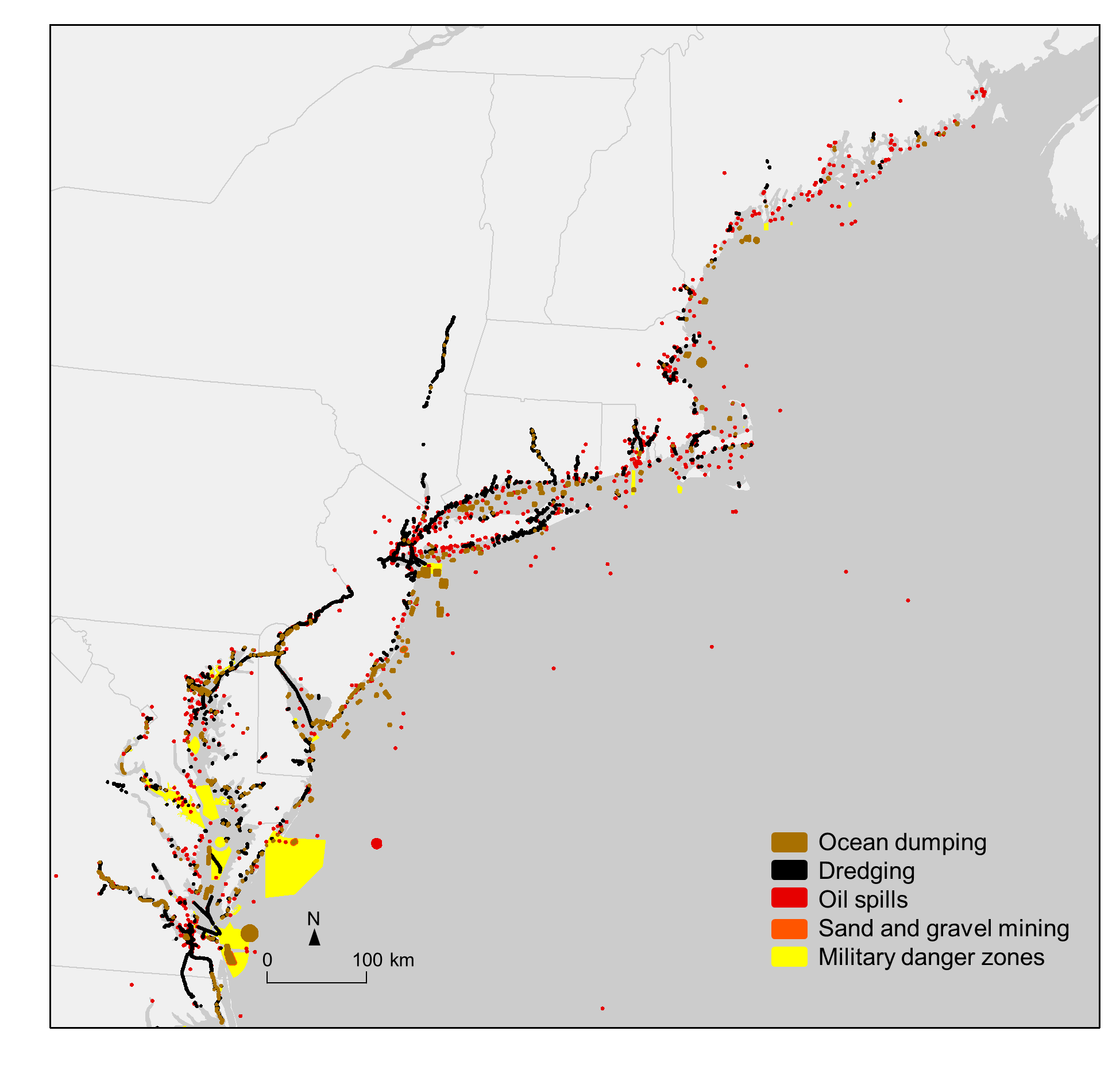
S1. Fig E. Military danger zones, ocean dumping, sand and gravel mining, and oil spills.**

We map ocean dumping, mining, and oil spills using a variety of sources. We map ocean dumping by selecting for permitted and active ‘disposal sites’ ocean uses [19], which has full Atlantic coverage (Fig E, Table C in S1). We map ocean mining using current (2015) federal sand and gravel lease data [20] from the national Marine Cadastre and select for lease areas (i.e. polygons) with active permits (i.e. lease expiration date beyond 2016) and where construction has started (Fig E, Table C in S1). To map ocean pollution, we use data from the U.S. Coast Guard Marine Casualty and Pollution Database and subset for oil pollution incidents greater than 10 gallons investigated by the U.S. Coast Guard since 2002 [21]. We use the volume of the oil spill to estimate the area affected by assuming oil would spread to a thickness of 0.001mm [30](Fig E, Table C in S1).

Tourist activities include kayaking, recreational boating, SCUBA diving, surfing, and wildlife viewing (Fig F in S1). To map these uses, we aggregate and merge data from the NEOD and MARCO portals [10,22]. NEOD data cover the entire Northeast, while MARCO recreational use data from surveys are reported for each Mid-Atlantic state (Delaware, Maryland, New Jersey, New York, and Virginia). These surveys identify general use and dominant areas for a variety of activities and we focus on dominant areas of use here. For kayaking, we buffer board and paddle events from the Northeast by 3km following Kappel et al. [1] and then merge with dominate Mid-Atlantic areas (i.e. polygons) for recreational kayaking, non-motorized vessels, and board and rowing from each state (Table C in S1). For recreational boating, we merge the upper quartile of recreational boater density from the Northeast with dominant areas for charter and personal recreational boating from each Mid-Atlantic state. We select only the upper quartile of boater density in the Northeast in order to align with the dominant areas identified in the Mid-Atlantic (Table C in S1). To represent the extent of SCUBA diving, we merge recreational SCUBA diving areas in the Northeast with polygons identified in the Mid-Atlantic states as the dominant areas for charter diving and snorkeling, recreational dive fishing, and SCUBA and snorkeling diving (Table C in S1). To represent surfing, we buffer stand up paddle board and surf races in the Northeast by 3km (following Kappel et al. [1]) and merge them dominant padding and surface water sports areas from the Mid-Atlantic states (S1 Table 3). Finally, to represent wildlife viewing, we merge commercial whale watching areas in the Northeast with dominant areas for charter, offshore, and recreational wildlife viewing, and areas for charter scenic viewing from the Mid-Atlantic states (Table C in S1). For wildlife viewing, we apply the whale-watching vulnerability scores used off the coast of Massachusetts [1]. We do not account for variable intensity for any of the tourist activities.

**
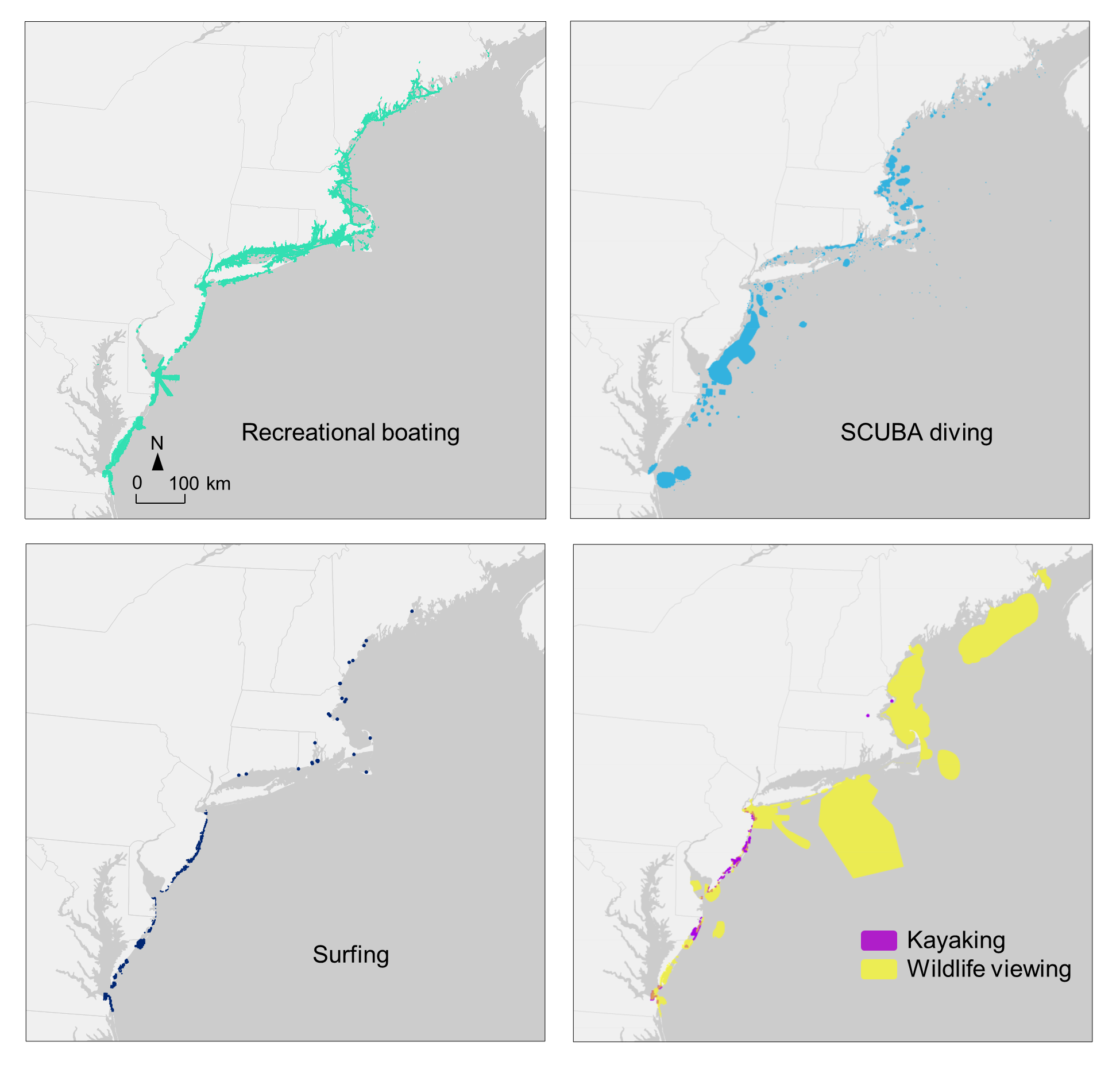
S1. Fig F. Tourist activities.**

#### Land-based stressors

We include and map land-based impacts including power plants, light pollution, inorganic pollution, and nutrient export into coastal waters (Fig G in S1). To map power plants, we use the coastal energy sites available on the MARCO portal [23], which cover the entire Atlantic, and buffer each plant by 3km following protocols from Massachusetts [1](Fig G in S1). Coastal power plants affect coastal ecosystems by entraining larvae and small plants as they draw in water to cool the plants [1] (Table C in S1). To map light pollution, we extract NOAA’s Nighttime Lights [24] for the Atlantic coastline and use the ArcGIS viewshed tool to project light intensity to the adjacent 32km offshore [24,31] (Fig G in S1); we choose 32km based on the outer extent of impact from light pollution [31]. We use the quartiles of modeled light intensity to define variable intensities of impact based on the methodology outlined above (Table C in S1).

**
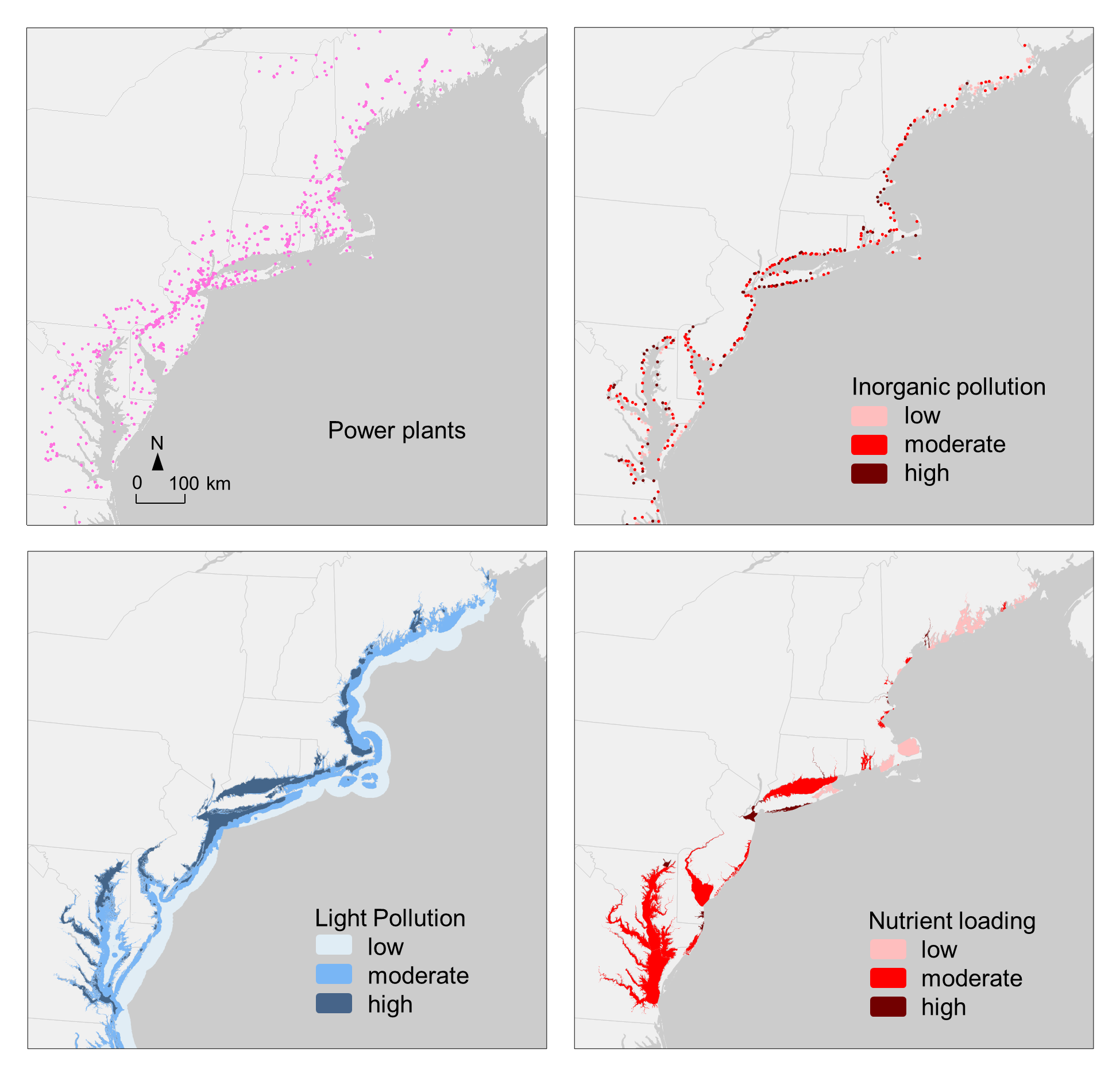
S1. Fig G. Land-based stressors.** Light, inorganic, and nutrient pollution are broken into three categories of intensity. Inorganic pollution is assigned to coastal pour points, and nutrient pollution is by estuary.

To estimate inorganic pollution we use the amount of impervious surface as a proxy for non-point source pollution [32]. This is a widely used approach to capture the effect of cars, roads, and urban areas on coastal habitats [1]. We use the National Land Cover Database (NLCD) and USGS hydrologic units [25,32] to calculate the percentage of each watershed comprised of impervious surfaces (Table C in S1). We model 408 coastal pour points in the study area, which uniquely identify independent upstream watersheds that range from large watersheds that span hundreds of square kilometers to small coastal watersheds that cover only a few HUC-12 hydrologic units [25]. We buffer each pour point by 3km, assign the relative amount of inorganic pollution delivered by the upstream watershed, and then classify the intensity of this stressor using the methodology outlined above.

In the absence of observed datasets with continuous coverage through our study area [33], we chose a modeled approach to estimate concentrations of nutrients in coastal estuaries. The USGS conducted assessments of the total nitrogen and phosphorous loading into coastal estuaries in the Northeast and Mid-Atlantic using the SPARROW model [26] , an empirical model that relates downstream observed nutrient loading to a parsimonious set of upstream predictors [34]. While this analysis is currently being redone by the USGS to update with newer methods and data, the latest analysis with full geographic coverage that we use here relies on older land cover classes (2002) for non-point source pollution estimation and point-source loading. Estimates are provided in kg/yr of total nitrogen and phosphorous for 43 major coastal estuaries or “estuary groups,” neighboring estuaries that are hydrologically and geomorphologically similar. To define these watersheds spatially, we use information from the EPA Estuary Data Mapper project that has defined 344 estuarine systems in the U.S.; we then cross-reference these to estuaries in Moore et al. [34].

We summarize nutrient estimates from the SPARROW modeling in Moore et al. [34] by estuary, not by tributary, leading us away from the approach common in the cumulative impacts literature of using diffusive modeling of point source loads, and towards estimating nutrient concentrations based on annual nutrient load and estuary water volume. Both approaches ignore advective processes that govern intra-annual movement of nutrients throughout the estuary and microbial denitrification due to an insufficient understanding and characterization of these processes at the scale of this study. Our concentration estimates are therefore best considered as indicators of relative loading between estuaries and not as estimates of the average annual concentration of total nitrogen and phosphorous in receiving waters. Water volume was calculated for each estuary using bathymetric data from NOAA’s NCEI [7] (Table C in S1).

#### Climate Change

While multiple metrics are important for capturing the effect of climate change on coastal and ocean habitats, we explore the effects of climate change by mapping the relative rate of increasing sea surface temperatures (Fig H in S1). We focus on sea-surface temperature because it affects all included habitat classifications, these effects are well documented and understood relative to other measures of climate change (e.g., increasing UV light), and the spatial data are readily available. Using Kappel et al. [1] for guidance, we calculate temperature anomalies using NOAA’s International Comprehensive Ocean-Atmospheric Datasets [27], which includes monthly means and standard deviations on a 1° grid. We first calculate the means and standard deviations from 1960 to 2000 for each coordinate within our area of interest. For each monthly data point from 2000 to 2015, we calculate the difference between the recorded temperature and the long-term mean; in all cases current temperatures were warmer than historical means. We then count anomalies as those temperature differences greater than the standard deviation. Areas with a greater number of anomalies represent areas warming at a faster rate. We use the inverse-distance weighted technique in ArcGIS to extrapolate temperature increases from the grid cell points into a continuous surface across the area of interest (Fig H in S1). We would ideally include a continuous range of warming SST, but the model requires vector based inputs so we further classify warming intensity within the area of interest. Areas for which the number of anomalies were in the bottom quartile were given the lowest intensity ranking; areas for which the number of anomalies were in the middle two quartiles were given a moderate intensity ranking; and those areas for which the number of anomalies was in the top quartile were given a high intensity ranking (Table C in S1). This results in a relatively coarse estimate of the variation in warming (i.e. only three intensities of warming), but allows for the impact of rising sea temperatures to be included across the area of interest.

**
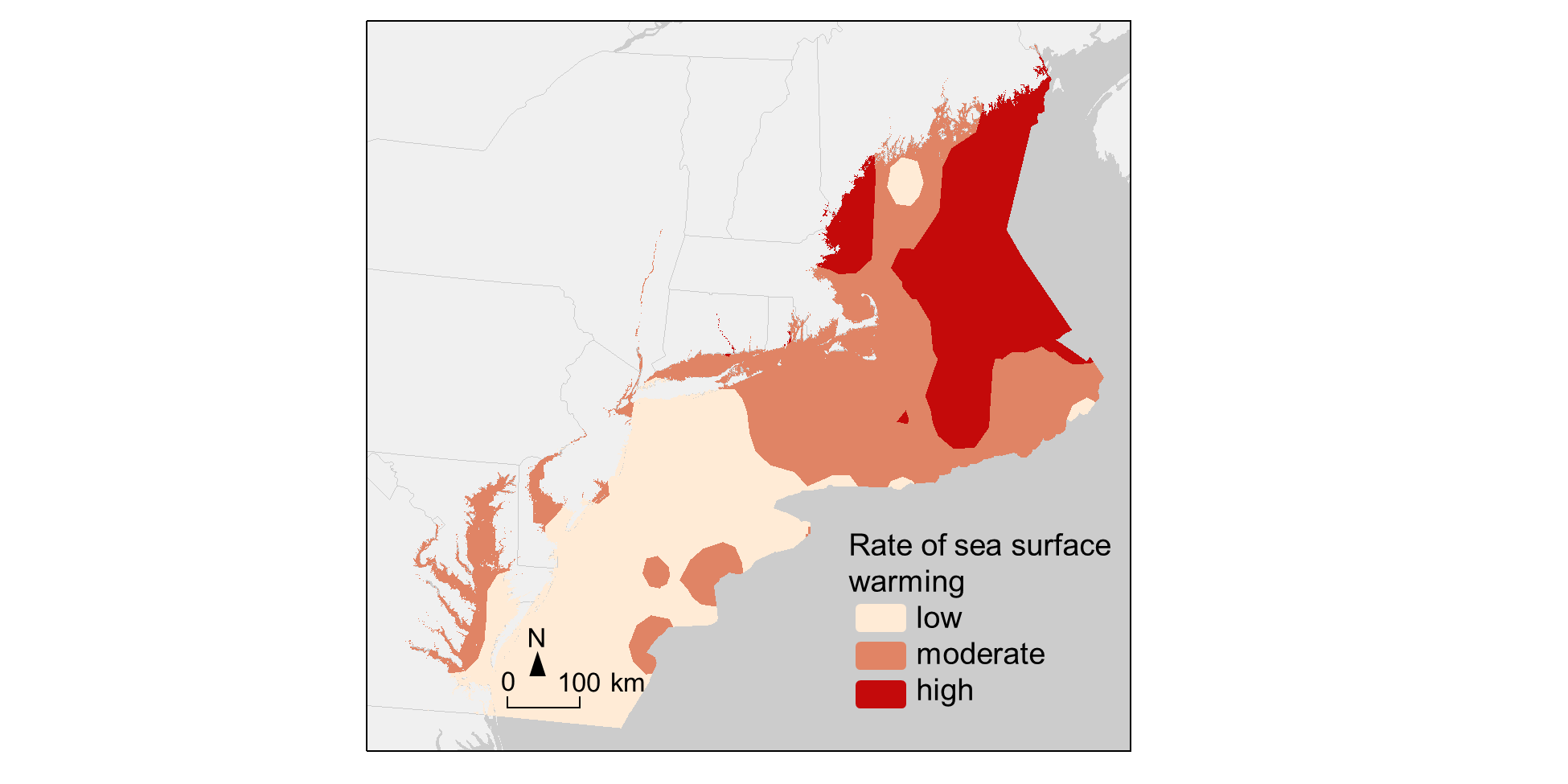
S1. Fig H. Increasing sea surface temperature.**

## Wind energy analysis

We map the *levelized cost of energy*, i.e. the cost of producing a unit (kilowatt hour) of energy, using the InVEST offshore wind energy model [35,36]. The levelized cost of energy is a useful metric for comparison across different energy generation technologies, but also serves as a more accurate proxy for social welfare than net present value in this context as it does not reflect economic rents that accrue in this thin market. As a result, it is more appropriate for public policy decisions when comparing against externalities associated with wind energy siting. We parameterize the model using a wind farm configuration of 40 turbines, each with a 5 megawatt capacity as an example design; this configuration is popular in Europe and it is unclear currently what farm configurations are going to become common in the U.S. Other model parameters for the U.S. east coast are adapted from Griffin et al. [35], including a 12.65% weighted average cost of capital, and costs of $10.5 (USD$ 2012) million per turbine and $2.6 million per foundation. Other values are the model defaults in InVEST for the 5.0 MW turbine [37].

The model output is a raster of the estimated levelized cost of energy. We convert this to points, then edit to remove points within shipping lanes (from NOAA Office of Coast Survey) and points within MPAs whose “protection focus” is ecosystems according to NOAA’s MPA Inventory [38,39]. A shapefile of active BOEM lease areas was acquired from BOEM’s website to identify these sites in the figure [40].

## References

1. Kappel CV, Halpern BS, Napoli N. Mapping cumulative impacts of human activities on marine ecosystems. Boston, MA: SeaPlan; 2012. Report No.: 03.

2. 10m-physical-vectors | Natural Earth [Internet]. Natural Earth; Available: http://www.naturalearthdata.com/downloads/10m-physical-vectors/

3. Environmental Sensitivity Index (ESI) Maps and GIS Data [Internet]. NOAA Office of Response and Restoration; Available: https://response.restoration.noaa.gov/maps-and-spatial-data/download-esi-maps-and-gis-data.html

4. The National Wetlands Inventory [Internet]. U.S. Fish and Wildlife Service; Available: https://www.fws.gov/wetlands/nwi/

5. Eelgrass [Internet]. Northeast Ocean Data Portal; Available: http://www.northeastoceandata.org/eelgrass/

6. Seagrass [Internet]. NOAA Office for Coastal Management; Available: https://marinecadastre.gov/data/

7. U.S. Coastal Relief Model [Internet]. NOAA National Centers for Environmental Information; 1999. Available: https://www.ngdc.noaa.gov/mgg/coastal/crm.html

8. Continental Margin Mapping (CONMAP) sediments grainsize distribution for the United States East Coast Continental Margin [Internet]. U.S. Geological Survey; Available: https://catalog.data.gov/dataset/continental-margin-mapping-program-conmap-sediments-grainsize-distribution-for-the-united-state06df8

9. Kappel CV, Halpern BS, Selkoe KA, Cooke RM. Eliciting Expert Knowledge of Ecosystem Vulnerability to Human Stressors to Support Comprehensive Ocean Management. In: Perera AH, Drew CA, Johnson CJ, editors. Expert Knowledge and Its Application in Landscape Ecology. Springer New York; 2012. pp. 253–277. doi:10.1007/978-1-4614-1034-8_13

10. Recreation [Internet]. Northeast Ocean Data Portal; Available: http://www.northeastoceandata.org/data/data-download/

11. Human Dimensions, Aquaculture [Internet]. Northeast Ocean Data Portal; Available: http://www.northeastoceandata.org/data/data-download/

12. Energy and Infrastructure [Internet]. Northeast Ocean Data Portal; Available: http://www.northeastoceandata.org/data/data-download/

13. Liquefied Natural Gas Import/Export Terminals [Internet]. U.S. Energy Information Administration; Available: https://www.eia.gov/maps/layer_info-m.php

14. LandScan [Internet]. Oak Ridge National Laboratory’s; Available: http://web.ornl.gov/sci/landscan/

15. Maritime [Internet]. Mid-Atlantic Ocean Data Portal; Available: http://portal.midatlanticocean.org/data-catalog/maritime-industries/

16. Marine Transportation [Internet]. Northeast Ocean Data Portal; Available: http://www.northeastoceandata.org/data/data-download/

17. Wrecks and Obstructions [Internet]. NOAA National Ocean Service; Available: https://marinecadastre.gov/news/load.php?url=posts/wrecks-and-obstructions.html

18. Danger Zones and Restricted Areas in the United States as of July 2015 [Internet]. NOAA Office for Coastal Management; Available: https://catalog.data.gov/dataset/danger-zones-and-restricted-areas-in-the-united-states-as-of-july-2012/resource/edcba640-2f61-48aa-b843-1b0893a1d903

19. Ocean Disposal Sites [Internet]. Marine Cadastre; Available: https://marinecadastre.gov/data/

20. Federal Outer Continental Shelf (OCS) Sand and Gravel Borrow Areas (Lease Areas). In: Data.gov [Internet]. [cited 25 Feb 2017]. Available: https://catalog.data.gov/dataset/federal-outer-continental-shelf-ocs-sand-and-gravel-borrow-areas-lease-areas

21. Marine Casualty and Pollution Database - Injury for 2002 - 2015 [Internet]. U.S. Coast Guard; Available: https://catalog.data.gov/dataset/marine-casualty-and-pollution-data-for-researchers

22. Recreation [Internet]. Mid-Atlantic Ocean Data Portal; Available: http://portal.midatlanticocean.org/data-catalog/recreation/

23. Coastal Energy Facilities [Internet]. Environmental Protection Agency; Available: http://portal.midatlanticocean.org/data-catalog/energy/#layer-info-coastal-energy-facilities

24. Version 4 DMSP-OLS Nighttime Lights Time Series [Internet]. NOAA National Centers for Environmental Information; Available: https://ngdc.noaa.gov/eog/dmsp/downloadV4composites.html#AVSLCFC

25. Hydrologic Unit Maps [Internet]. U.S. Geological Survey; Available: https://water.usgs.gov/GIS/huc.html

26. USGS SPARROW Surface Water-Quality Modeling [Internet]. U.S. Geological Survey; Available: https://water.usgs.gov/nawqa/sparrow/

27. International Comprehensive Ocean-Atmosphere Data Set (ICOADS) [Internet]. NOAA; Available: http://icoads.noaa.gov/

28. DePiper GS. Statistically Assessing the Precision of Self-reported VTR Fishing Locations. NOAA Northeast Fisheries Science Center; p. 16. Report No.: 229.

29. New York’s offshore wind energy development potential in the great lakes: Feasibility study. New York State Energy Research and Development Authority; 2010 Apr. Report No.: 10-04.

30. Patin S. Oil fate during oil spills in the marine environment [Internet]. Available: http://www.offshore-environment.com/oil.html

31. Verutes GM, Huang C, Estrella RR, Loyd K. Exploring scenarios of light pollution from coastal development reaching sea turtle nesting beaches near Cabo Pulmo, Mexico. Glob Ecol Conserv. 2014;2: 170–180. doi:10.1016/j.gecco.2014.09.001

32. Homer C, Dewitz J, Yang L, Jin S, Danielson P, Xian G, et al. Completion of the 2011 National Land Cover Database for the conterminous United States-Representing a decade of land cover change information. Photogramm Eng Remote Sens. 2016;81: 345–354.

33. National Monitoring Network for U.S. Coastal Waters and Tributaries. In: National Monitoring Network of the National Water Quality Monitoring Council [Internet]. [cited 25 Feb 2017]. Available: https://acwi.gov/monitoring/network/

34. Moore RB, Johnston CM, Smith RA, Milstead B. Source and Delivery of Nutrients to Receiving Waters in the Northeastern and Mid-Atlantic Regions of the United States1. JAWRA J Am Water Resour Assoc. 2011;47: 965–990. doi:10.1111/j.1752-1688.2011.00582.x

35. Griffin R, Chaumont N, Denu D, Guerry A, Kim C-K, Ruckelshaus M. Incorporating the visibility of coastal energy infrastructure into multi-criteria siting decisions. Mar Policy. 2015;62: 218–223. doi:10.1016/j.marpol.2015.09.024

36. Griffin R, Buck B, Krause G. Private incentives for the emergence of co-production of offshore wind energy and mussel aquaculture. Aquaculture. 2015;436: 80–89. doi:10.1016/j.aquaculture.2014.10.035

37. Sharp R, Tallis H, Ricketts T, Guerry A, Wood SA, Chaplin-Kramer R, et al. InVEST [Internet]. The Natural Capital Project, Stanford University, University of Minnesota, The Nature Conservancy, and World Wildlife Fund; 2016. Available: http://www.naturalcapitalproject.org/software/

38. U.S. Marine Protected Areas Boundaries: MPA Inventory [Internet]. [cited 18 Sep 2017]. Available: https://nmsmarineprotectedareas.blob.core.windows.net/marineprotectedareas-prod/media/archive/pdf/helpful-resources/inventory/mpa_inventory_2014_metadata.pdf

39. Shipping Fairways, Lanes, and Zones for US waters [Internet]. NOAA Office of Coast Survey; 2015. Available: https://inport.nmfs.noaa.gov/inport-metadata/NOAA/NOS/OCS/inport/xml/39986.xml

40. Atlantic OCS Lease Status Information | BOEM [Internet]. [cited 28 Sep 2017]. Available: https://www.boem.gov/Atlantic-OCS-Lease-Status-Information/
